# Exploring Serum Transferrin Regulation of Nonferric Metal Therapeutic Function and Toxicity

**DOI:** 10.3390/inorganics8090048

**Published:** 2020-08-29

**Authors:** Josué A. Benjamín-Rivera, Andrés E. Cardona-Rivera, Ángel L. Vázquez-Maldonado, Christian Y. Dones-Lassalle, Héctor L. Pabón-Colon, Héctor M. Rodríguez-Rivera, Israel Rodríguez, Jean C. González-Espiet, Jessika Pazol, Jobaniel D. Pérez-Ríos, José F. Catala-Torres, Marielie Carrasquillo Rivera, Michael G. De Jesus-Soto, Nicolle A. Cordero-Virella, Paola M. Cruz-Maldonado, Patricia González-Pagan, Raul Hernández-Ríos, Kavita Gaur, Sergio A. Loza-Rosas, Arthur D. Tinoco

**Affiliations:** 1 Department of Chemistry, University of Puerto Rico, Río Piedras Campus, Río Piedras, PR 00931, USA); 2 Departamento de Química y Bioquímica, Facultad de Ciencias e Ingeniería, Universidad de Boyacá, Tunja 150003, Colombia

**Keywords:** serum transferrin, endocytosis, nonferric metal ions, metal transport, bioavailability, bioactivity, metal therapeutic function and toxicity

## Abstract

Serum transferrin (sTf) plays a pivotal role in regulating iron biodistribution and homeostasis within the body. The molecular details of sTf Fe(III) binding blood transport, and cellular delivery through transferrin receptor-mediated endocytosis are generally well-understood. Emerging interest exists in exploring sTf complexation of nonferric metals as it facilitates the therapeutic potential and toxicity of several of them. This review explores recent X-ray structural and physiologically relevant metal speciation studies to understand how sTf partakes in the bioactivity of key non-redox active hard Lewis acidic metals. It challenges preconceived notions of sTf structure function correlations that were based exclusively on the Fe(III) model by revealing distinct coordination modalities that nonferric metal ions can adopt and different modes of binding to metal-free and Fe(III)-bound sTf that can directly influence how they enter into cells and, ultimately, how they may impact human health. This knowledge informs on biomedical strategies to engineer sTf as a delivery vehicle for metal-based diagnostic and therapeutic agents in the cancer field. It is the intention of this work to open new avenues for characterizing the functionality and medical utility of nonferric-bound sTf and to expand the significance of this protein in the context of bioinorganic chemistry.

## 1. Introduction

Serum transferrin (sTf) is an 80 kDa glycoprotein that is part of the transferrin family of proteins [1]. It is present at 30–60 μM in blood and plays the major role of maintaining iron (Fe) homeostasis within the body [2]. As part of this role, it partakes in numerous regulatory functions. By binding Fe(III) upon release into the bloodstream, it prevents the hydrolysis and precipitation of the metal ion [3] thus increasing its blood solubility to micromolar levels and, consequently, facilitating its bioavailability. STf also inhibits the reduction of Fe(III) to Fe(II), which if otherwise left uncontrolled would lead to Fe toxicity from excessive production of reactive oxygen species (ROS). By having a very high affinity for the metal ion, it also participates in regulating bacteriostasis by controlling the Fe(III) uptake by bacteria and preventing its scavenging by pathogenic ones. STf is the principal molecular route by which Fe is delivered to cells throughout the body via a transferrin receptor 1 (TfR) recognition pathway that results in cellular uptake of the metal via endocytosis.

**Figure 1.**
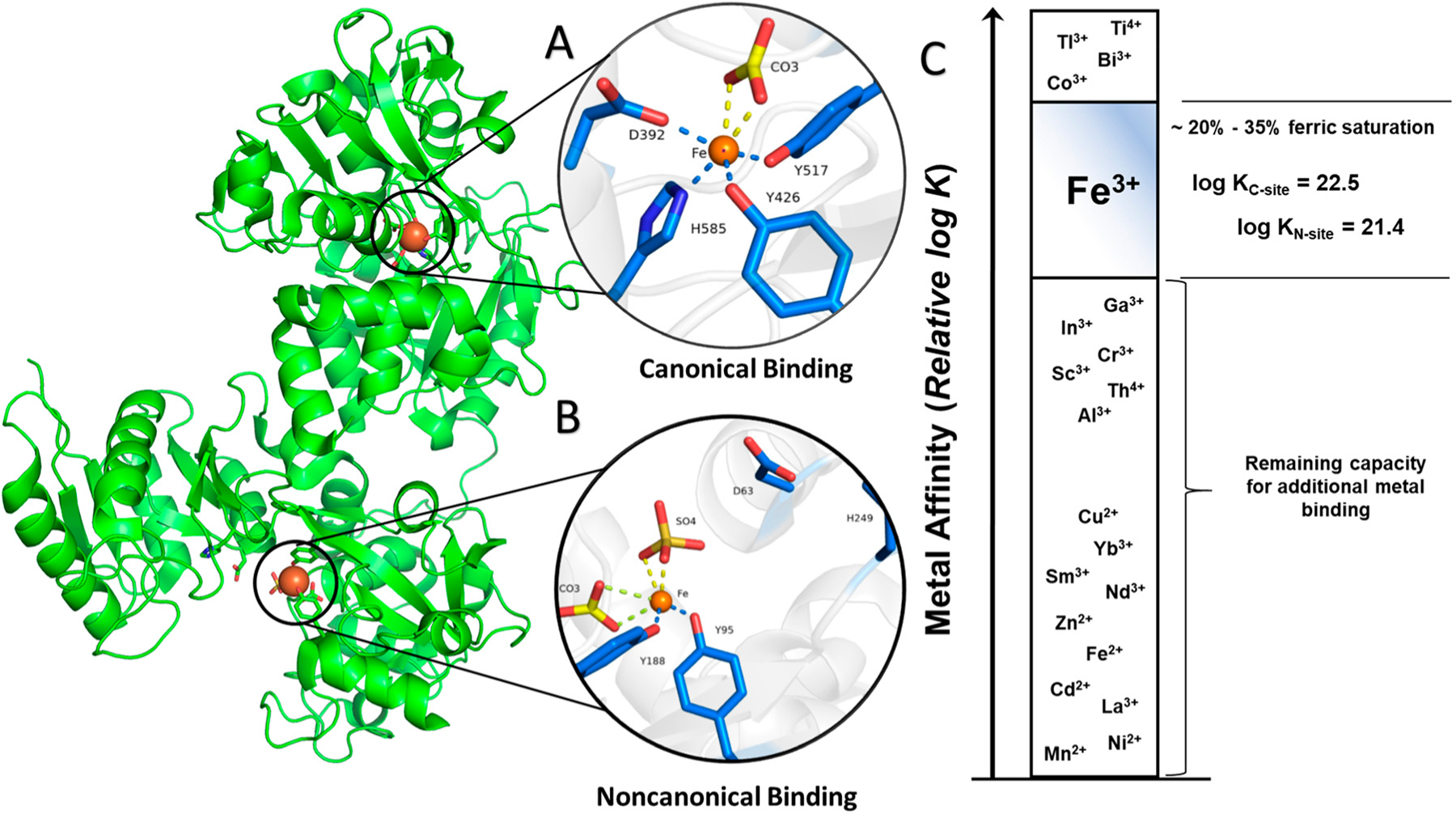
Canonical (closed, (**A**)) and noncanonical (open, (**B**)) Fe(III) binding to serum transferrin (sTf). (**C**) Representation of sTf metal affinity that suggests the capacity for additional nonferric metal binding. The relative positions were taken from experimental values in Ref. [4,8] and references within. PDB code: 3QYT.

Structurally, sTf is primed for Fe(III) binding. It consists of two lobes defined by its N and C terminus. Both lobes are divided into two subdomains that come together to form metal binding sites, one per lobe, each able to bind one Fe(III) ion with very high affinity (log K_C-site_ = 22.5 and log K_N-site_ = 21.4) [4]. From the perspective of the moieties that directly coordinate the Fe(III) in vivo (the primary sphere of coordination), the metal binding sites are identical. They consist of four protein amino acids; two tyrosines, one histidine, and one aspartic acid. The remainder of the Fe(III) coordination sit is fulfilled by a bidentate carbonate anion (CO_3_^2−^), which is referred to as the synergistic anion. This anion, in the form of bicarbonate (HCO_3_^−^), is prevalent in our blood at 27 mM and serves as a buffering agent. It synergizes with sTf to stabilize Fe(III) binding in blood, providing for a 6-coordinate environment that is favorable for Fe(III) [5]. This physiologically relevant coordination of Fe(III) is referred to as the canonical modality of metal binding (Figure 1A). Several carboxylate containing ligands can substitute for the carbonate and retain the bidentate coordination [6] while still fulfilling the canonical modality. The order of magnitude difference in the Fe(III) affinity to both sites is due to differences in intramolecular interactions within the secondary sphere of coordination. STf undergoes a major conformational change upon Fe(III) binding. Each lobe transitions from an open to a closed conformation, which stabilizes the protein and alters its surface charge. These changes are believed to be essential for recognition by the TfR and its mediation of cellular uptake. Recent crystal structures have demonstrated that Fe(III) can bind in a non-canonical modality (Figure 1B) in which Fe(III) is bound by alternate protein residues and synergistic anions or molecules that result in semi-open or open lobe conformations [7]. These structures are believed to be physiologically relevant and represent different coordination modes in the multi-step pathway from the Fe(III) uptake in blood to, ultimately, cellular release.

**Figure 2.**
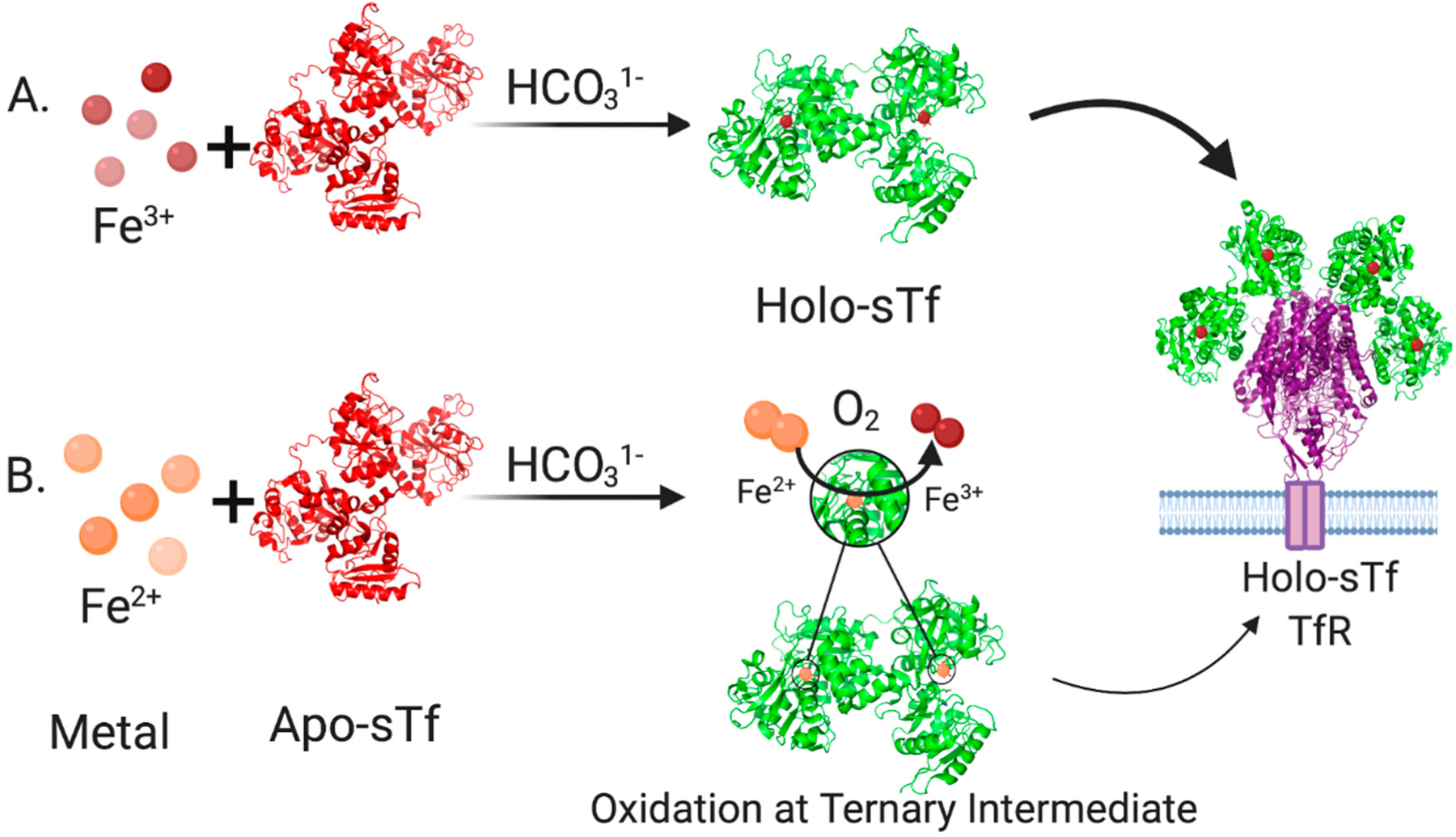
Pathways for sTf binding of Fe forming the Holo-sTf complex that is recognized by the transferrin receptor 1 (TfR). (**A**). In the most common pathway, Fe(III) released into blood is immediately bound by sTf in synergism with bicarbonate. (**B**). In a less common pathway, Fe(II) leached into blood is bound by sTf in synergism with bicarbonate. STf can operate as a ferroxidase and oxidize Fe(II) to Fe(III). Created with BioRender.com.

In human blood, the speciation of sTf consists of 39.2% metal free(apo)-sTf, 11.2% Fe_C_-sTf, 22.9% Fe_N_-sTf, and 26.7% Fe_2_-sTf (also referred to as holo-sTf) [9]. That sTf is nearly 30% Fe(III)-saturated has long been implicated as an indicator of the capacity of sTf to bind nonferric metal ions. Extensive metal binding studies have been performed to evaluate the general metal binding capacity of sTf. A correlation was established by Sadler et al., between the affinity of metal ions for the hydroxide ion (a measure of their Lewis acidity) and their affinity for the first site of sTf (log K_1_) [4]. The higher the affinity for the hydroxide ion, the higher the affinity for sTf (Figure 1C). This suggests that sTf has a preference for binding hard Lewis acidic metal ions [5], which Fe(III) is. While this correlation is generally true today, there are a few implicit discrepancies. Not all metal ions bind sequentially to the metal binding sites nor do they all bind in the canonical modality. Furthermore, the synergistic anion or molecule may not be the same for all metal ions nor actually be required. The correlation also does not imply the physiological relevance of the metal-sTf interaction in vivo nor rule out the possibility of interactions with metal ions that are expected to have a weak affinity. For instance, there is some evidence to suggest that sTf can bind Fe(II) that leaches into the bloodstream, providing for another Fe-based regulatory function of sTf. The presence of Fe(II) in serum is an indicator of a diseased state in which there is a loss of Fe-homeostasis. STf may be able to rescue the leached Fe(II) and rapidly convert it to Fe(III) via a ferroxidase like mechanism [10,11] (Figure 2).

Several lines of study have advocated for a role of sTf in regulating the bioactivity of nonferric metal ions [12,13]. The therapeutic function and toxicity of numerous metals have been linked to sTf but often in tenuous ways, focusing exclusively on the binding interactions between them, and in a subset of studies, a binding interaction between the metal-bound sTf complexes and the TfR. In this review article, we assess physiologically relevant structural and speciation studies to understand how sTf binds nonferric metal ions and to determine the features of the nonferric-sTf complexes that enable a favorable interaction with the TfR to trigger endocytosis. In addition, we evaluate the requirements for the release of nonferric metals into the cytosol and the connection between this release and their impact on human health, using in vivo data where available. We also consider how Fe(III)-bound sTf plays a pivotal in the manifestation of nonferric metal ion transport and bioavailability. A specific focus is made on non-redox active hard Lewis acidic metals. Finally, we evaluate studies dedicated to engineering sTf as a delivery agent for metal-based diagnosis and therapeutic strategies for cancer applications. This work enriches and deepens the understanding of a protein that has long been thought to be very well understood.

## 2. Structural Characterization of sTf and Its Fe(III) Binding

**Figure 3.**
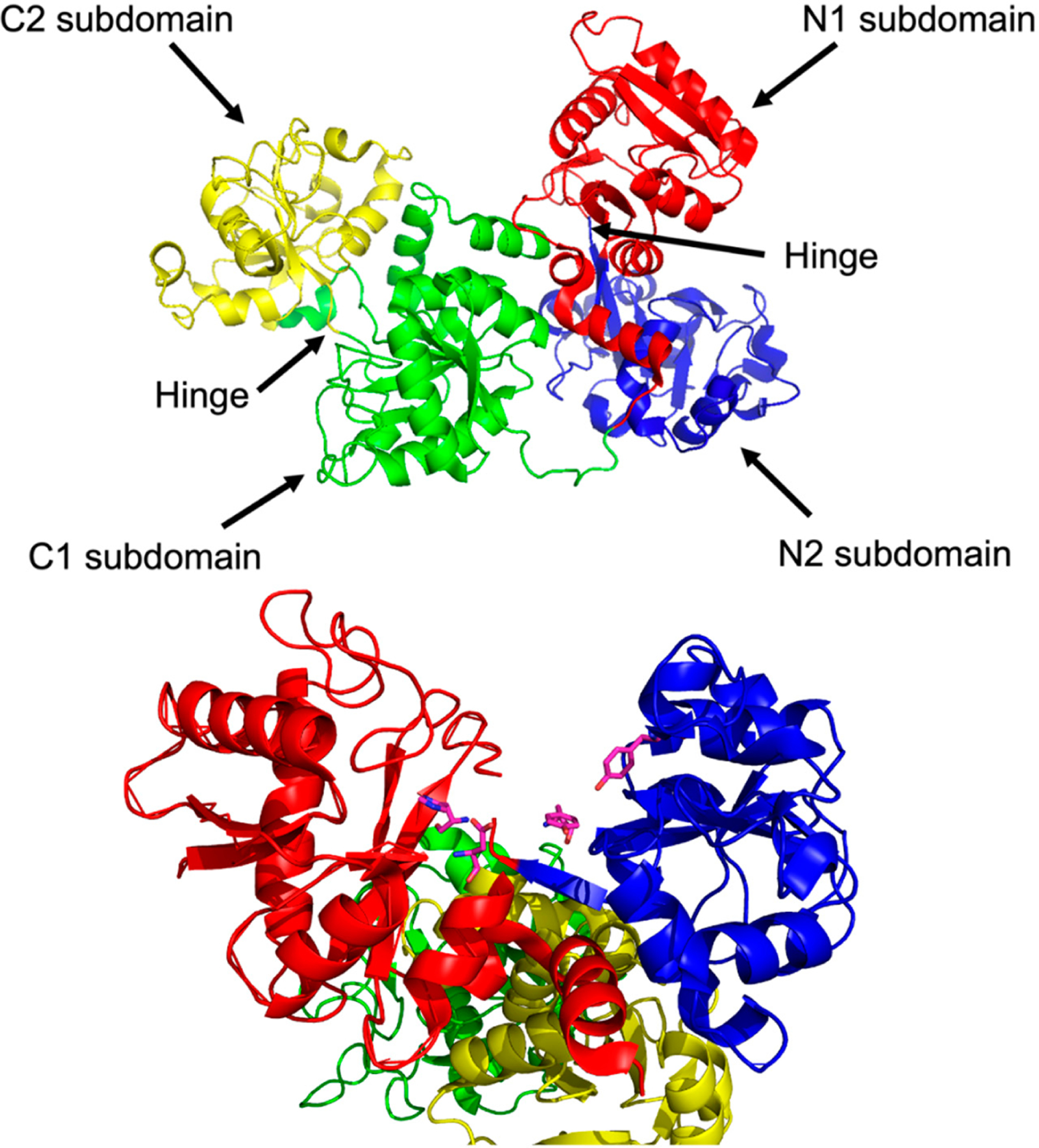
The structure of glycosylated apo-sTf divided into its lobes and subdomains (Top). A close up of the metal binding residues of the N-terminal domain (Bottom). PDB code: 2HAV.

To appreciate the interplay between structure and function, it is necessary to briefly survey structural details of sTf and TfR that are important for Fe(III) cellular delivery and, potentially, the delivery of other metals. STf is a bilobal protein that is believed to be part of an evolutionary gene duplication event from monolobal transferrins [14,15], which appears to have increased its Fe(III)-binding capacity and affinity [16]. It consists of 679 residues that are divided into the N-terminus lobe (N-lobe; residues 1–331) and the C-terminus lobe (C-lobe; residues 339–679) and they are held together by a linker peptide that contains residues 332–338 [17] (Figures 1 and 3). The lobes are subdivided into subdomains C1, C2, N1, and N2, which are connected by hinges [17]. The subdomains are structurally different. Subdomains C1 and N1 are discontinuous, meaning that the amino acid residues representative of each are not continuous, while C2 and N2 have a continuous polypeptide chain. Subdomain N1 is composed of residues 1–92 and 247–331, whereas subdomain N2 is composed of residues 93–246. Similarly, the C1 subdomain has residues 339–425 and 573–679 while subdomain C2 has residues 426–572 [17]. The subdomains form two Fe(III) binding sites involving identical coordinating moieties. The Fe(III) binding residues (Figure 1) include two tyrosines (Tyr95 and Tyr188 of N-lobe; Tyr426 and Tyr517 of C-lobe), a histidine (His249 of N-lobe; His585 of C-lobe), and an aspartic acid (Asp63 of N-lobe; Asp392 of C-lobe) in addition to a carbonate ion, which is notoriously known as the synergistic anion [6]. Altogether they coordinate Fe(III) with a coordination number 6 in the canonical modality. One tyrosine borders the N2 or C2 subdomains close to the hinges and the second tyrosine is located inside the N2 or C2 subdomains. The aspartic acid is within the N1 or C1 domains and the histidine is found in the N1 or C1 subdomain hinge [17]. The carbonate anion binds in a pocket containing an arginine residue (Arg124 of the N-lobe and Arg456 of the C-lobe).

**Figure 4.**
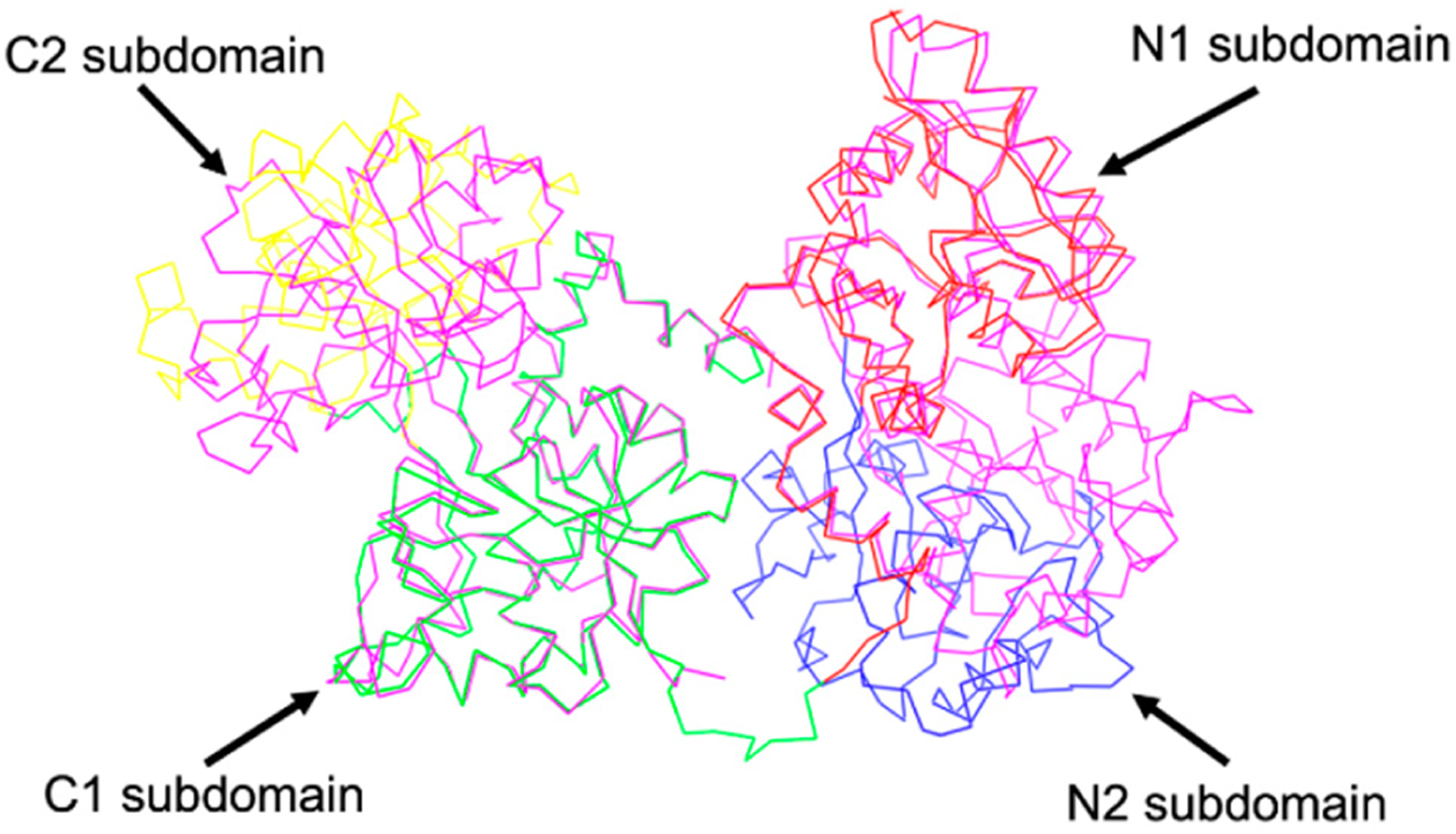
Sequence alignment of human apo-sTf and diferic pig sTf (magenta colored protein). PDB code for human apo-sTf: 2HAV, PDB code for diferric pig sTf: 1H76.

By superimposing their apo-sTf (Fe(III) free) structure with pig diferric sTf (Fe_2_-sTf), Wally et al., found that the N1 and C1 subdomains align very well, while the C2 and N2 subdomains do not, which indicates that the N2 and C2 subdomains are responsible for opening and closing the lobes to capture and release the iron (Figure 4). The apo form is categorized by an open-conformation and the Fe(III) bound form is categorized by a closed-conformation. The reality is that apo-sTf exists in an equilibrium between an open and closed-conformation but with the closed-conformation existing less than 10% of the time [18]. In the apo form, the C-lobe is open to a hinge angle 49.5° and the N-lobe is open to 59.4°. When observing the secondary structure of the hinge regions, there is a difference between the N and C lobes [17]. The N-lobe hinge is formed of longer strands consisting of two antiparallel β-sheets compared to the C-lobe hinge, which only has one β-sheet shorter than the same sheet in the N-lobe and contains the hinge in an unstructured region. This suggests that the N-lobe has more flexibility and could contribute to the difference in Fe(III) affinity and binding and release kinetics.

**Figure 5.**
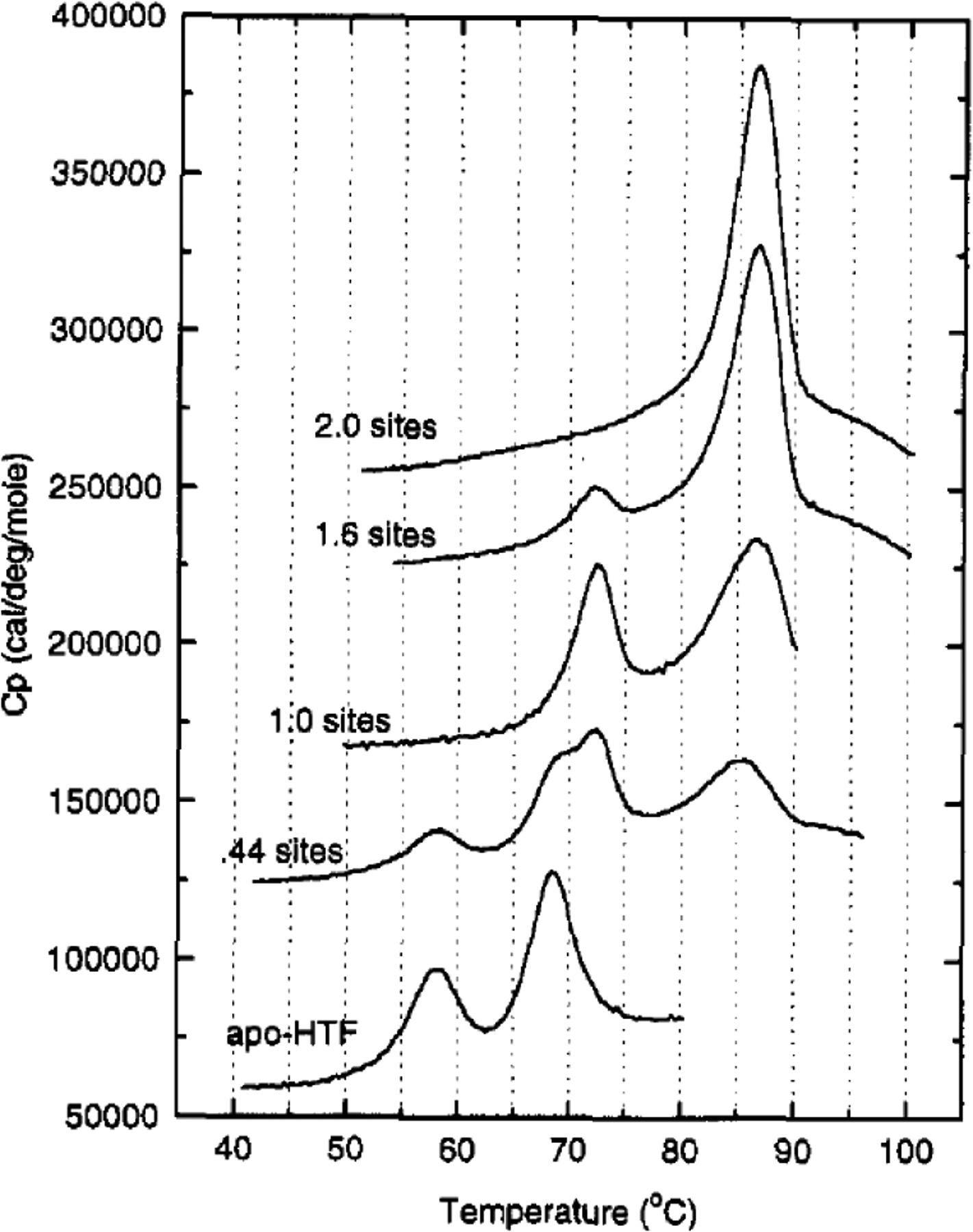
Differential scanning calorimetry (DSC) scans of 20 μM sTf with different equivalents of Fe(III). Reprinted with permission from [18]. Copyright (1994) American Chemical Society.

**Figure 6.**
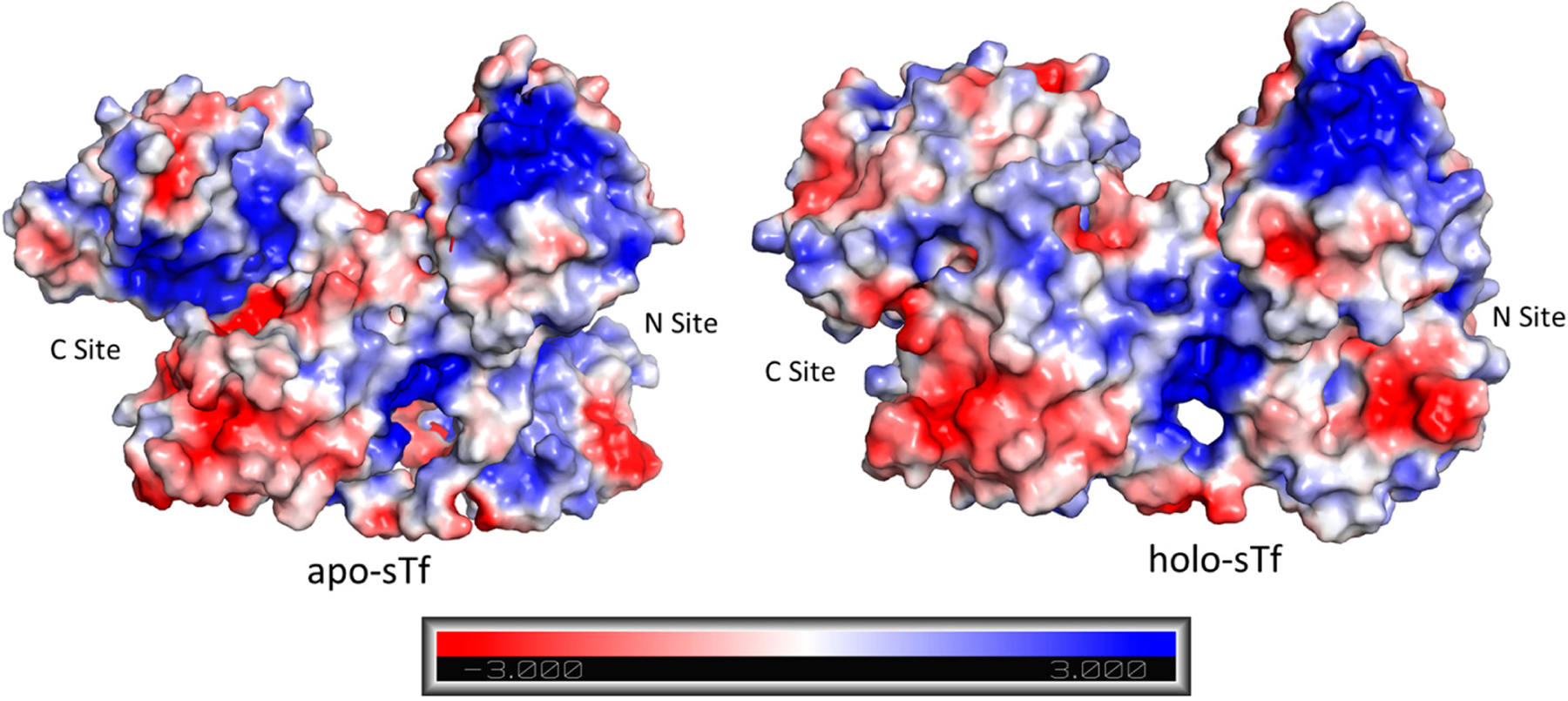
Distribution of the surface charge for apo-sTf (PDB code: 2HAU) and for holo-sTf (Fe_2_-sTf) (PDB code: 3V83). The blue color represents positively charged regions and the red represents negatively charged regions.

A cooperative effect in the binding of Fe(III) exists between the sTf lobes, in which the binding of Fe(III) in one lobe helps strengthen the binding in the other lobe. This physicochemical property could be observed in a differential scanning calorimetry (DSC) experiment performed by Lin et al. that monitored the titration of Fe(III) into apo-sTf (Figure 5) [19]. Under the experimental conditions used with Fe(NTA)_2_ as the source of Fe(III), the metal ion binds sequentially to the binding sites, first to the C-site and then to the N-site, as confirmed by urea-polyacrylamide gel electrophoresis (PAGE) [20,21]. In the DSC study, Fe(III) binding to the C-site caused a huge 29.4 °C increase of the melting temperature (T_m_) of the C-lobe, indicative of a major stabilization owed to the associated conformational change [19]. The N-lobe, without any bound Fe(III), also experienced a not insignificant T_m_ increase of 5 °C. After the second Fe(III) bound to the N-site, the N-lobe T_m_ increased an additional 13.6° [19]. Wally et al. noted that the interface of the lobes contain hydrophobic patches [17]. The hydrophobic interactions may cause for movement in one lobe as the other one closes due to Fe(III) binding. Furthermore, Asp240 and Arg308 residues in the N2 and N1 subdomains, respectively, can form salt bridges with the Arg678 and Asp376 residues in the C1 domains [17]. Yajima et al. found by calculation of electrostatic potentials in and out of the molecule that the surface charge in Fe_2_-sTf is more negative than apo-sTf revealing that the electrostatics of the protein are altered due to Fe(III) binding. By performing a similar analysis between the apo-sTf structure (PDB code: 2HAU) and Holo-sTf structure (PDB code: 3V83), the same conclusion is reached (Figure 6) [22]. This altered surface charge of the protein is key for the binding process with the TfR [22] and thus electrostatics may drive the onset of endocytosis. Furthermore, the development of key electrostatic interactions are fundamental to the heightened stability of the N- and C-lobes following Fe(III) binding especially in the second sphere of Fe(III) coordination. Near the N-site, Lys296 and Lys206 engage in a hydrogen bonding interaction due to a single proton dissociation between the two amino acids [23,24]. Near the C-site, Lys534, Arg632, and Asp634 form an electrostatic triad [25]. The dilysine interaction and electrostatic triad help to maintain the protein’s closed conformation.

## 3. STf Interaction with TfR and Cellular Delivery of Fe(III) by Endocytosis

**Figure 7.**
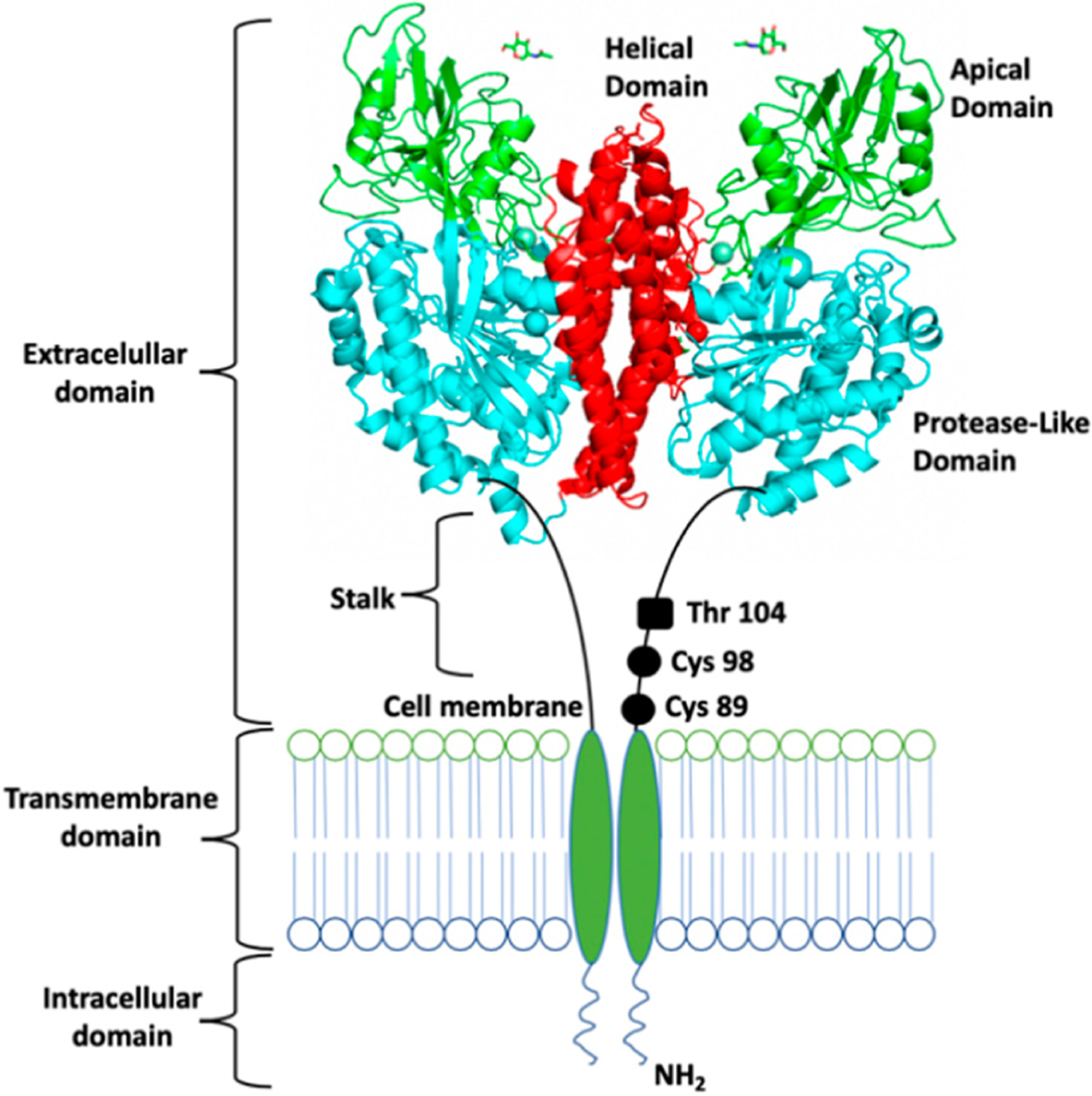
Schematic representation of the TfR. Figure shows the extracellular domain, with the stalk and the three subdomains (helical domain, apical domain, and protease-like domain), the transmembrane domain, and the intracellular domain. PDB code: 1CX8.

Although the human TfR can vary depending on cell type and Fe_2_-sTf affinity, it maintains structural consistency [26,27]. It is a 180 kDa homodimer type II transmembrane glycoprotein found on the cell membrane surface [28]. Its monomers, of approximately 769 residues each, are linked by two disulfide bridges [27]. Each monomer contains three major domains (Figure 7): (1) an ectodomain (or a C-terminal extracellular domain), (2) a transmembrane domain, and (3) an intracellular N-terminal domain [28]. The ectodomain is composed of 671 residues made up of two main subunits: the domain’s head, which has a globular butterfly-shaped conformation and the 29 Å stalk (approximately 37 residues long) that separates this head from the transmembrane domain. The ectodomain is subdivided into three subdomains: apical, helical, and protease-like domain [28]. It is specifically where the linkage between sTf occurs. The transmembrane domain consists of 20–28 residues that create a hydrophobic region. It contains palmitoylation sites, specifically at Cys 62 and Cys 67, which help TfR to cling to the cell membrane [29]. The intracellular N-terminal domain is made up of approximately 61–66 residues.

**Figure 8.**
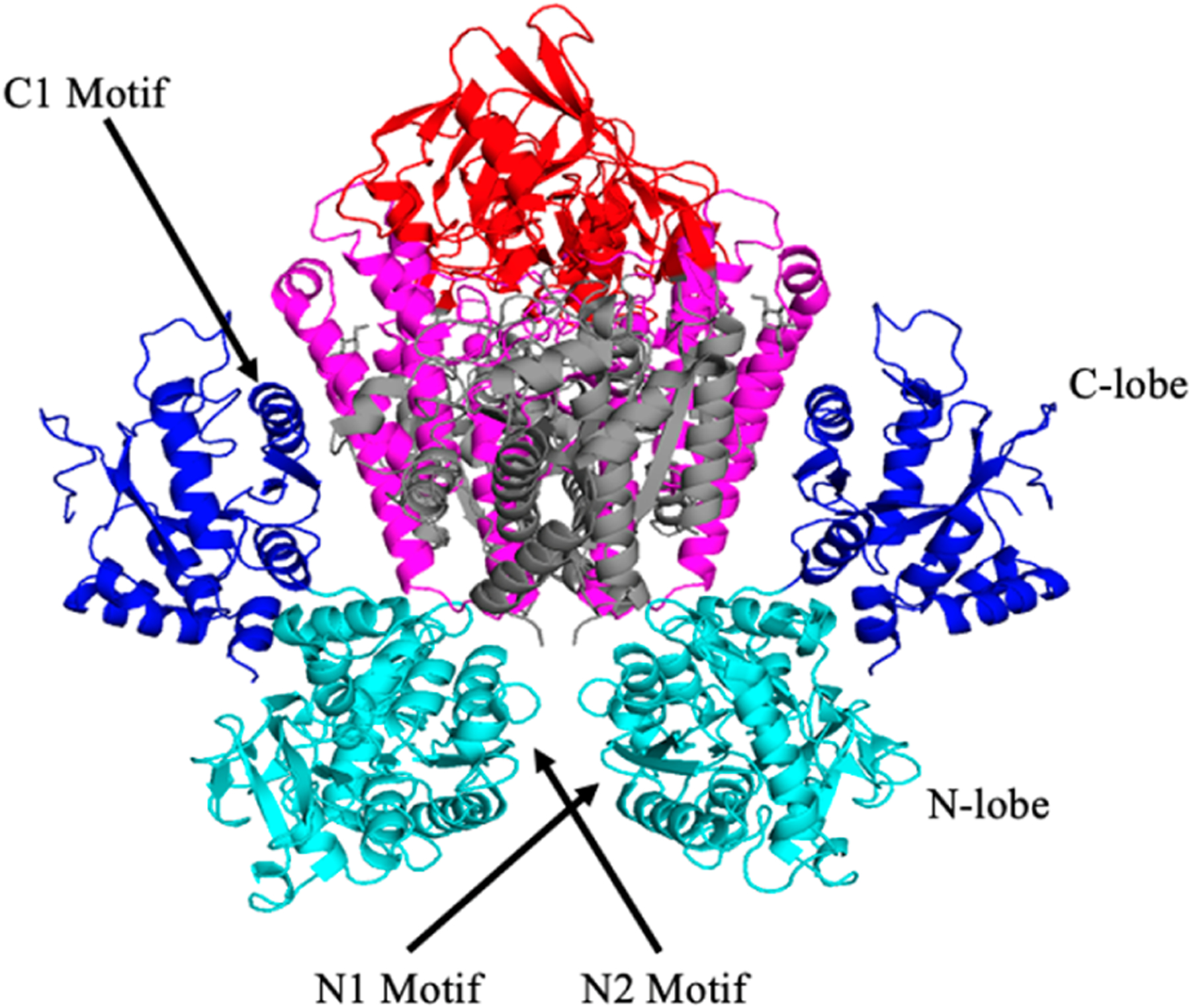
The structure of the Fe_N_-sTf-TfR complex and location of the different motifs, C-lobe and N-lobe. PDB code: 3S9M.

Due to its dimeric nature, the ectodomain can bind two sTf molecules. Cryo electron microscopy (cryo-EM) was initially used by Cheng et al. to understand the points of contact between the two [27]. They found that Pro142, Arg143, Lys144, and Pro145 of the N2 subdomain and Tyr71, Leu72, Ala73, and Pro74 of the N1 subdomain interact with the TfR ectodomain residues Leu122, Tyr123, Trp124, Asp125, Asn662, and Glu664. They also determined that the C1 lobe residues His349, Arg352, Leu353, Asp356, Glu357, Ser359, Val360, Glu367, Glu369, Ser370, and Glu372 are in proximity of the helical domain of TfR and the corresponding residues Leu619, Arg623, Arg629, Gln640, Try643, Arg646, Phe650, and Arg651. More recently the X-ray crystal structure of the Fe_N_-sTf-TfR complex (monoFe(III) at the N-site) was obtained at a resolution of 3.22 Å [30]. The structure provides greater molecular details of the interactions between sTf and TfR. Three different binding regions were analyzed: The sTf N1-TfR motif, the sTf N2-TfR motif, and the sTf C1-TfR motif (Figure 8) [30]. The difference between the two N-motifs is that the N2-motif lacks ionic bonds and has weaker interactions with the receptor. Due to their stronger interactions, the N1 and C1 motifs remain attached to the same α-helix in the TfR throughout the endocytosis cycle [30]. Conformational changes within the TfR ectodomain occur when Fe(III)-bound sTf binds. For instance, a translation of the apical and protease-like domains occurs, which causes a reorientation of the monomers in TfR. These conformational changes are likely responsible for the initiation of endocytosis for Fe(III) uptake into cells.

It is important to discuss sTf interaction with the TfR as both a pH dependent and Fe(III)-dependent process. The pH values of significance are pH 7.4 (blood pH) and pH 5.5 (endosomal pH following acidification). Leverence et al. performed studies to explore the interaction of apo-sTf, two monoferric sTf forms (Fe_C_-sTf and Fe_N_-sTf), and Fe_2_-sTf with TfR at pH values that approximate these physiologically relevant ones using electrospray ionization-mass spectrometry (ESI-MS) [31]. Near blood pH levels, Fe_2_-sTf exclusively formed the saturated sTf:TfR complex (2:1 ratio). The Fe_C_-sTf and Fe_N_-sTf complexes and apo-sTf could not saturate TfR but could nonetheless interact with the receptor. The apo-sTf finding was a surprise given that it was long-thought unable to bind to TfR at this pH. Its affinity was found to be weaker than the monoferric sTf proteins, which are readily outcompeted by Fe_2_-sTf for TfR binding. At endosomal pH, the trend is reversed, with apo-sTf having the strongest affinity to the receptor. This strong interaction is necessary for sTf to return to the cell membrane and be recycled for further Fe(III) uptake events.

**Figure 9.**
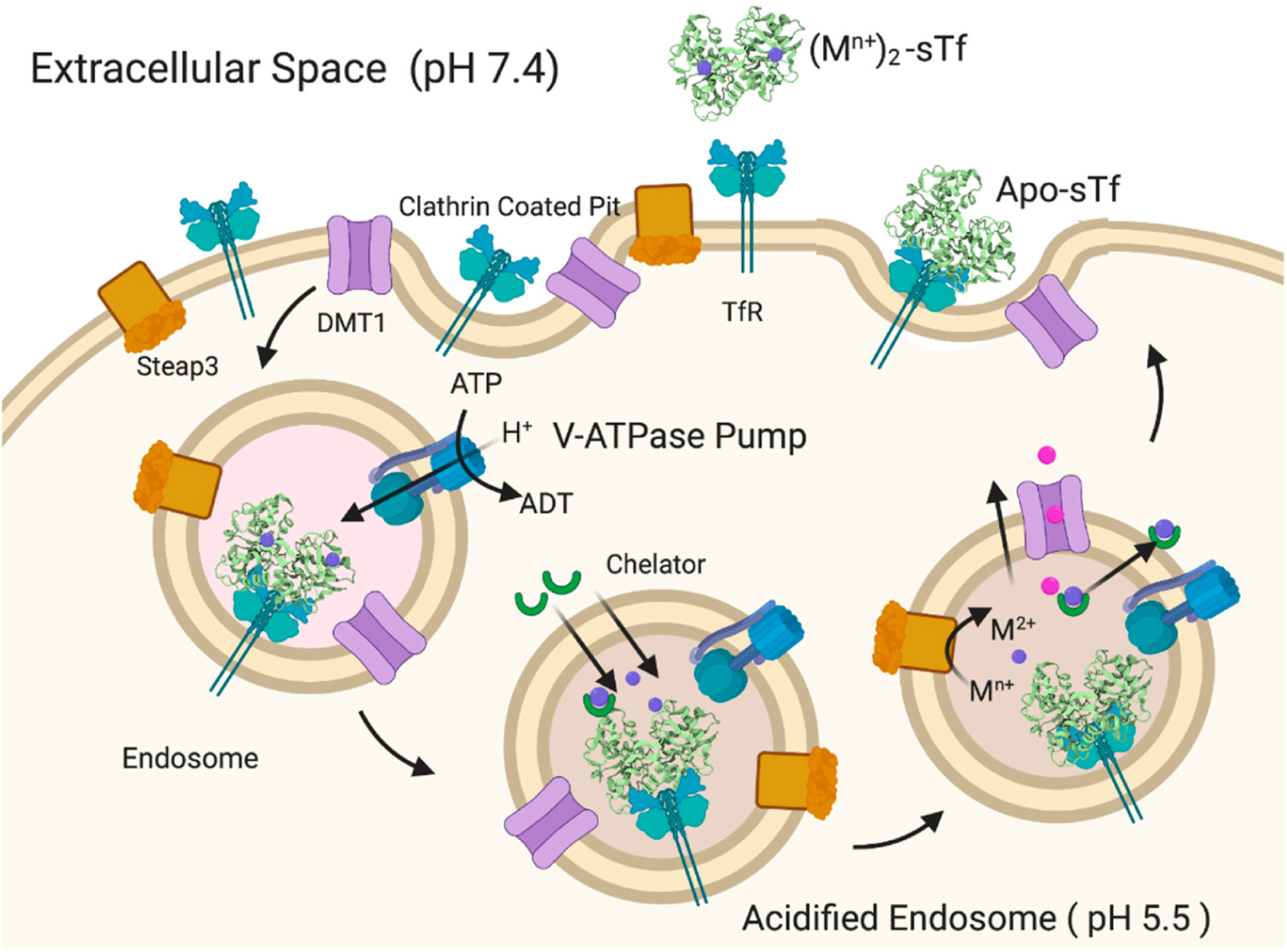
Generalized pathway for (M^n+^)_2_-sTf TfR-mediated endocytosis and metal ion release into the cytosol. Created with BioRender.com.

**Figure 10.**
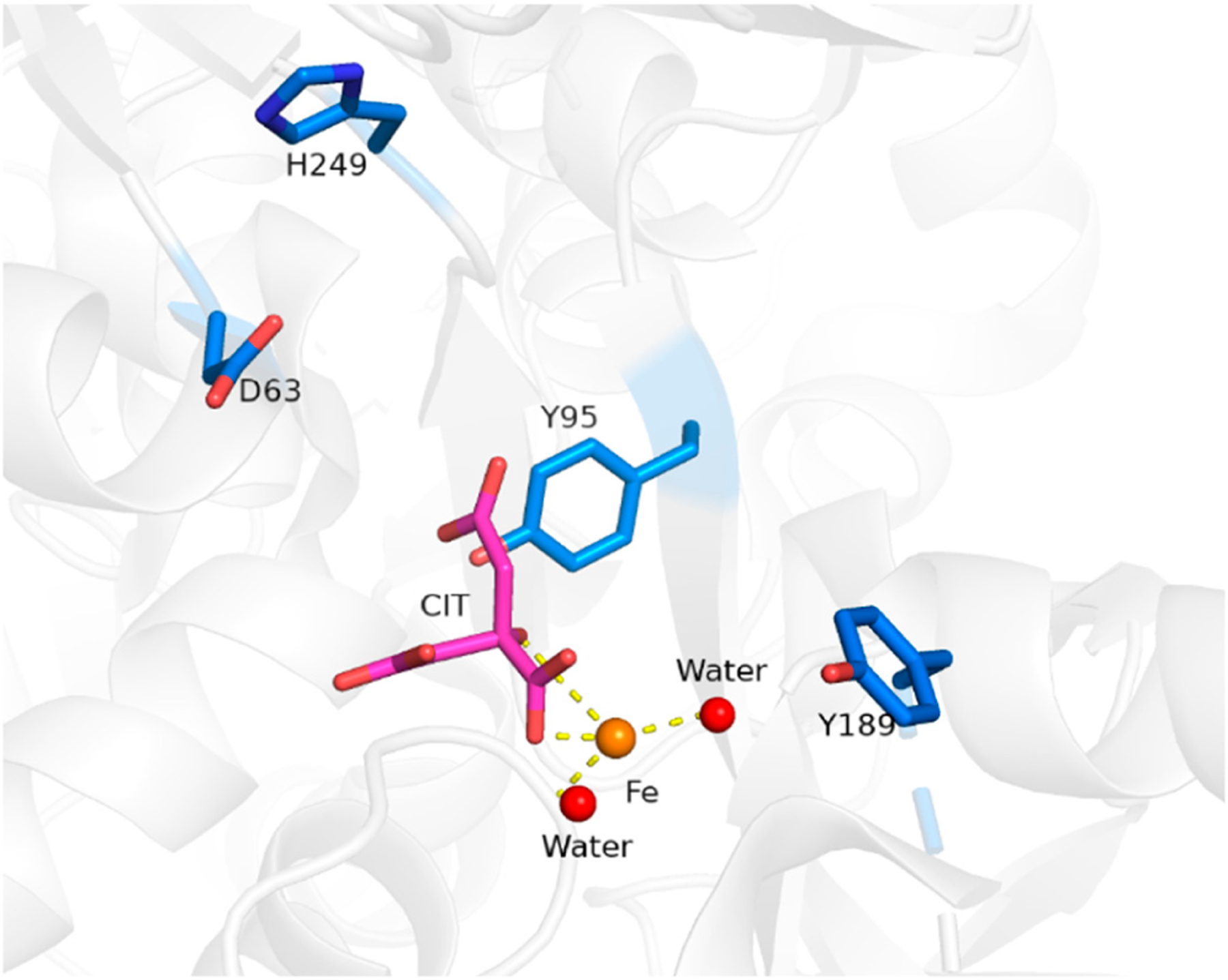
X-ray structure of citrate coordinated Fe(III) near the N-site of sTf. PDB code: 6JAS.

Fe_2_-sTf recognition by the TfR is essential to the initiation of endocytosis (Figure 9). This TfR-mediated process involves clathrin coating of the ensuing endosome to protect it from proteolytic degradation and to consequently protect the enclosed sTf and TfR so that they may be recycled. The adapter protein complex 2 (AP-2) mediates the formation of a proton-pumping endosome that includes several membrane proteins such as Steap3, a ferrireductase [32,33]. Once the endosome enters the cell, it is acidified to a pH of 5.5, regulated by vacuolar ATPase [34]. Although TfR-mediated endocytosis has been widely studied, there is not a consensus regarding the release of the Fe from sTf within the endosome and its delivery into the cytosol. It is generally accepted that the process involves acidification and reduction of Fe(III) to Fe(II). The debate stems from the order of the reduction event. One camp of researchers argues that acidification coupled with chelation by an intracellular chelator like citrate or adenosine triphosphate (ATP) results in dissociation of Fe(III) from sTf [35]. The metal ion then exists in a redox active, labile form, which is readily reduced by Steap3 to Fe(II). The divalent metal transporter 1 (DMT1) then transports Fe(II) into the cytosol where it transiently forms part of the labile iron pool (LIP) to be distributed for storage in ferritin and inserted into Fe-dependent proteins/enzymes for functionalization [36–40]. Another camp of researchers believes that the interaction between Fe_2_-sTf and TfR1 alters the redox properties of the bound Fe(III) by increasing its reduction potential from −0.53 to −0.30 V vs. NHE (Normal Hydrogen Electrode) so that it is within the biological window [41,42]. Steap3 is then able to directly reduce Fe(III) to Fe(II) [42]. This reduction event would significantly weaken the metal affinity, driving the log β value from 43.5 ((Fe^3+^)_2_-sTf) to 13 ((Fe^3+^)_2_-sTf) [43,44]. The metal ion could then undergo facile dissociation, possibly with the help of a chelator and be delivered out of the endosome by the DMT1. Lay et al. lend support to the molecular pathway involving direct reduction of Fe(III) within sTf bound to TfR1 but suggest that ascorbate is the likely reducing agent and citrate is the chelating agent [45]. This debate is not settled. Nonetheless, the acidification event in either route is likely important for improving the chelator affinity for the Fe(III) and fine tuning the metal ion’s redox potential. Acidification alone does not lead to Fe(III) dissociation as it was previously thought that the protonation of the dilysines in the N-site and of the electrostatic triad of the C-site could trigger an opening of the protein that would dispel the metal ion [23–25]. Instead, the acidification may enable the chelator to penetrate the metal binding site and induce a semi-open conformation that ultimately leads to metal release. Sun et al. has obtained an X-ray structure that captures Fe(III) bound in a semi-open conformation due to sulfate anion binding to the metal ion that prevents binding of the Asp and His residues [7] (Figure 1B). Sun et al. has also very recently obtained an X-ray structure (PDB code: 6JAS) that demonstrates citrate binding Fe(III) near the sTf metal binding site but without any metal ion covalent interaction with the canonical protein residues; a sort of snapshot of citrate scavenging the metal from sTf as Lay and others have proposed [45] (Figure 10).

## 4. Challenging the Perception of Structural Requirements for Metalated-sTf Endocytotic Uptakeinto Cells

**Figure 11.**
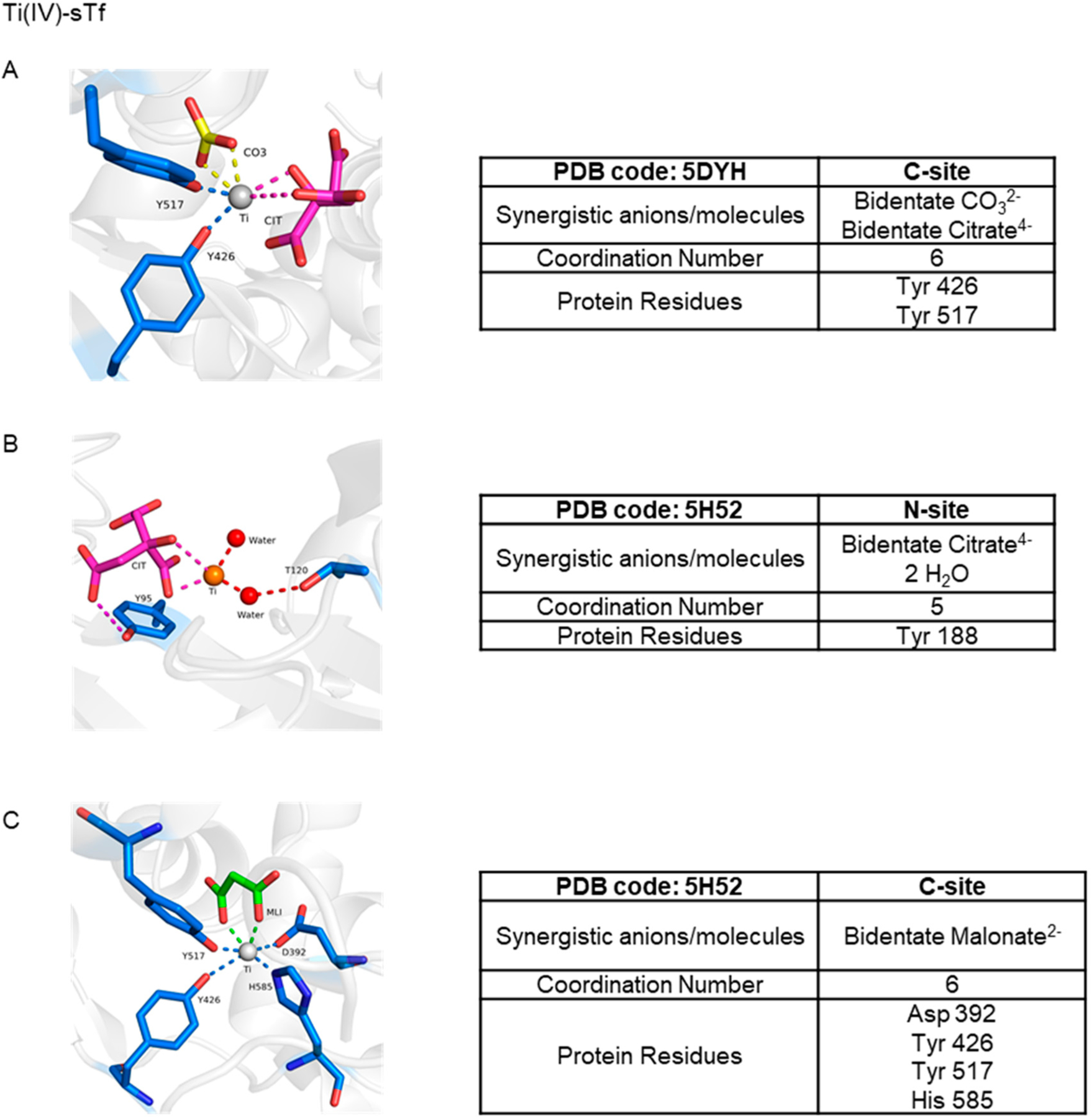
X-ray structures of Ti(IV) coordinated by sTf. Ti(IV) is observed bound in a semi-open conformation at the C-site (**A**) and the N-site (**B**) with citrate serving as a synergistic anion and in a closed conformation at the C-site (**C**) with malonate serving as a synergistic anion.

STf binding to the TfR alone is not enough for the induction of endocytosis as clearly apo-sTf, which can bind the TfR at pH 7.4 [31], is incapable of doing this. Some have argued that a metalated-sTf complex that exists in a closed conformation is required [46] for the appropriate TfR recognition and likely subsequent structural modification within the cell membrane, signaling for the initiation of endocytosis. This premise has been challenged by the finding that Ti(IV)-bound sTf, which exists in a noncanonical semi-open conformation [8,47], can be internalized by cells [8]. In the presence of blood levels of citrate (100 μM), sTf binds Ti(IV) at both tyrosines of the metal binding site and utilizes citrate and carbonate as synergistic anions producing a 6-coordinate complex (Figure 11A) [8]. The citrate coordination of the metal prevents binding of the Asp and His residues, resulting in a partial lobe closure. This Ti_2_-sTf complex binds with high affinity to the TfR (log K_A_ = 14.6) though not as strongly as the canonical Fe_2_-sTf complex (log K_A_ = 17.5) [48,49]. Ti(IV) is able to bind in several different modalities at the sTf metal binding sites but not all are expected to be physiologically relevant (Figure 11B,C).

**Figure12.**
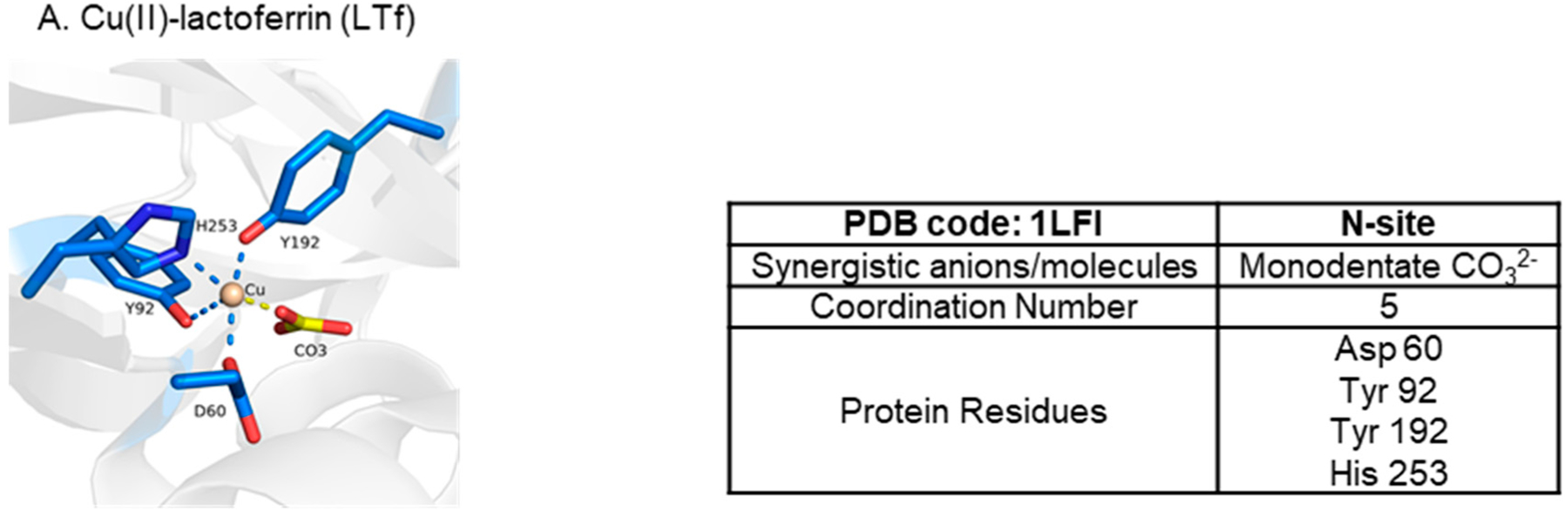

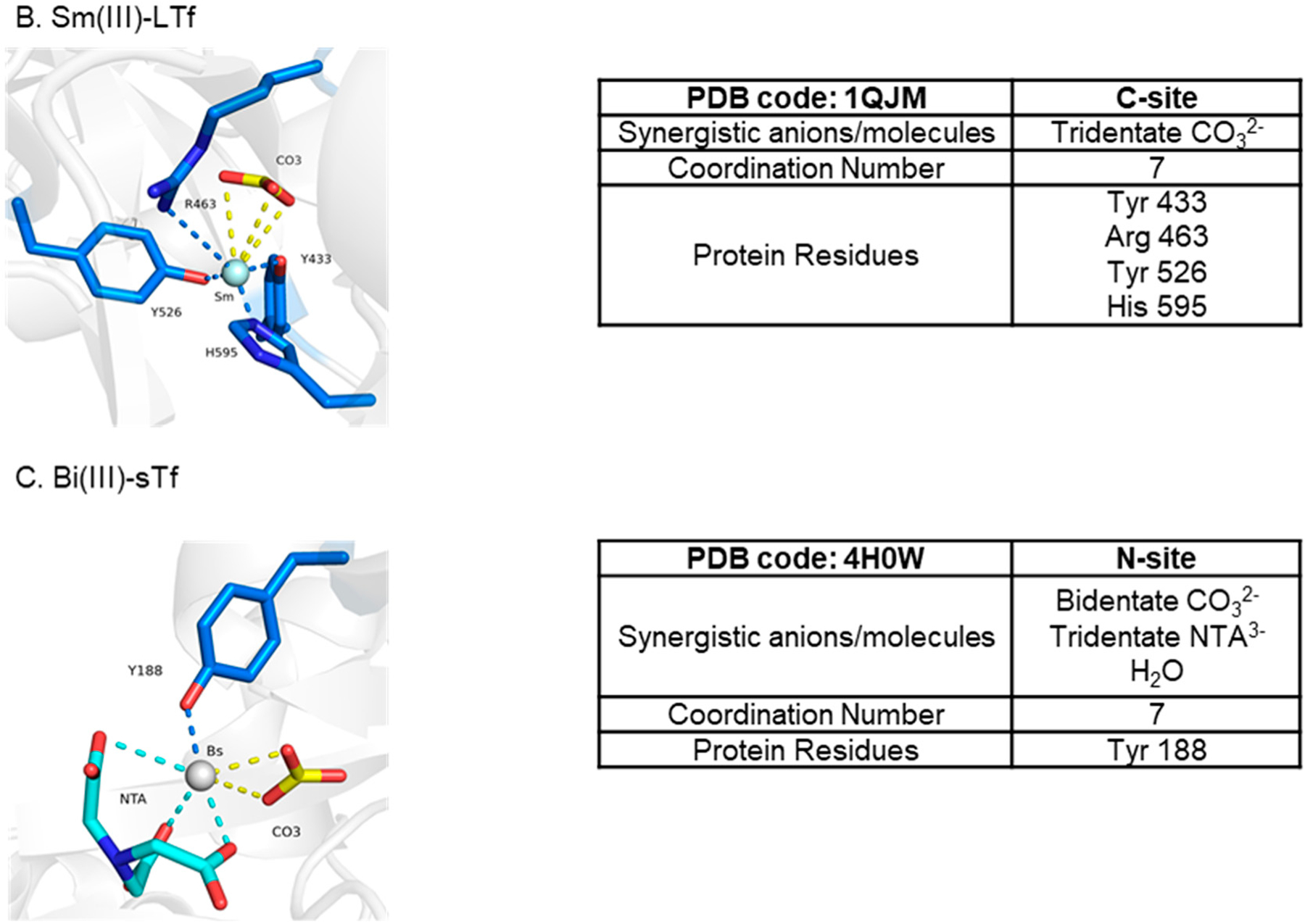
X-ray structures of sTf coordination of Cu(II) (**A**), Sm(III) (**B**), and Bi(III) (**C**) in noncanonical modality.

Ti(IV) is amongst several metal ions that have been structurally shown capable of binding at the metal binding site in a noncanonical modality (Figure 12) [7,50,51]. Copper(II) (Cu(II)), probably owed to the Jahn–Teller effect [5], can bind in a distorted square pyramidal form (coordination number 5) with the carbonate synergistic anion bound in a monodentate fashion [50] (Figure 12). This coordination modality is nonetheless a closed conformation. Unsurprisingly, bismuth(III) (Bi(III)) [7] and samarium(III) (Sm(III)) [51] have both been crystallographically captured in a semi-open conformation with a coordination number of 7 (Figure 12). Due to their placement in the periodic table (*n* = 6), these metal ions have much bigger 6-coordinate ionic radii (Bi(III)~1.1 Å and Sm(III) 1.02 Å) than high-spin Fe(III) (0.645 Å) [52] and are capable of forming complexes of a high coordination number [5]. The Bi(III) structure in Figure 12 is not a physiologically relevant one because of the non-endogenous NTA synergistic anion but it models the well-documented semi-open conformation that sTf binding of the metal ion produces [53]. The Sm(III) structure is likely of physiological relevance. Sm(III) is shown coordinated to carbonate in an unusual tridentate form and to four sTf residues with an Arg taking the place of Asp due to structural accommodation for the bigger metal ion (Figure 12). This noncanonical coordination enables a closed lobe conformation. Lanthanides and actinides are expected to be bound by sTf in a higher coordination number modality. A combination of quantum mechanical (QM) and molecular dynamics (MD) studies have predicted that sTf in synergism with carbonate and water molecules can bind curium(III) (Cm(III)) in a hepta-dentate modality, plutonium(IV) (Pu(IV)) in an octadentate or nonadentate modality, and thorium(IV) (Th(IV)) in an octadentate modality [54,55]. Surprisingly, the metal coordination is modeled with a closed lobe conformation.

An intriguing finding is that a high affinity of a metal ion to sTf does not correlate with a strong interaction of the metal-sTf complex to the TfR (Table 1). The discrepancy can be attributed to two possibilities. Gallium(III) (Ga(III)) represents one possibility. It is a non-redox active chemical mimic of Fe(III) that is capable of binding with similar affinity to biological ligands and potentially interfere with Fe-dependent processes [56]. It undergoes extensive hydrolysis in aqueous solution especially at pH 7.4 [57]. The propensity for Ga(III) hydrolysis is so high that although sTf can bind Ga(III) with high affinity (log K_1_ = 20.3) [58], Ga(III) hydrolysis can compete with sTf binding. The higher the concentration of Ga(III) in solution, the more it may distribute between sTf and hydrolyzed oxo/hydroxo species. This competitive interplay can affect the interaction of Ga(III)-bound sTf with the TfR as there may be a more open and closed conformation dynamic with Ga(III)-bound sTf.

**Figure 13.**
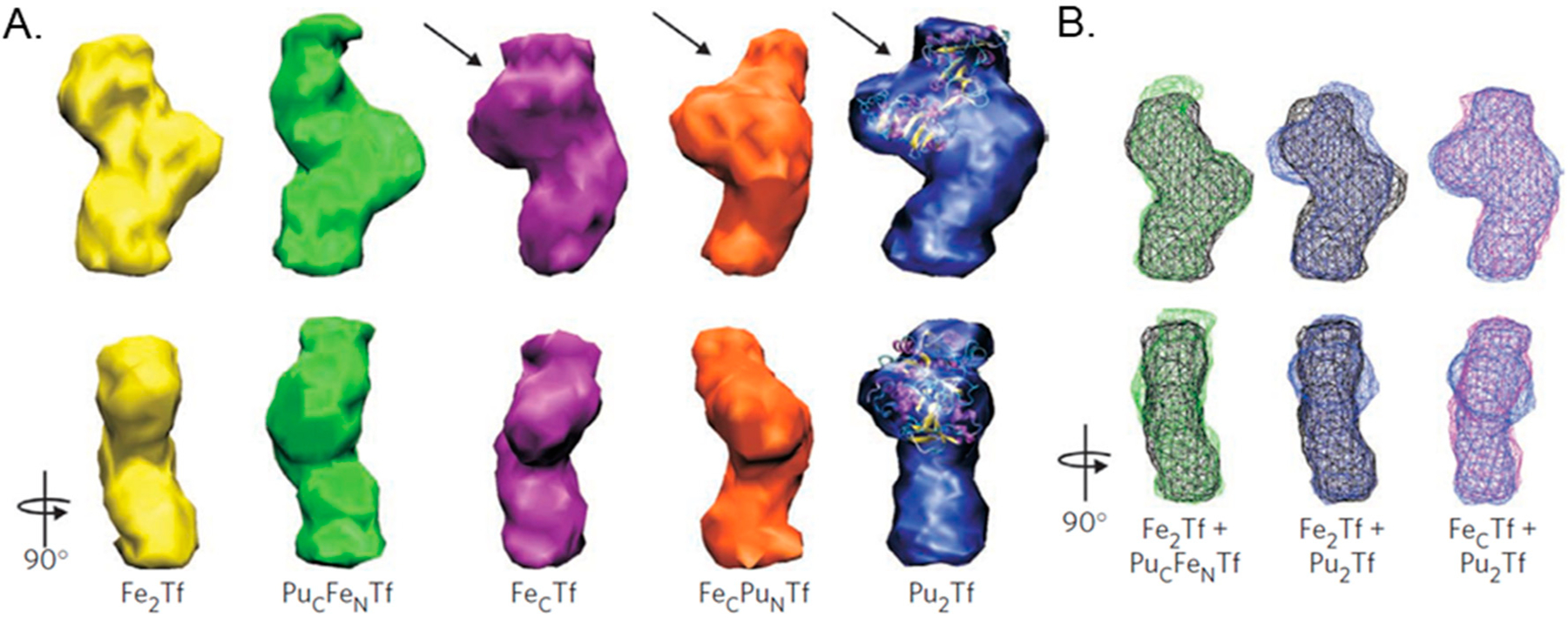
Structural models of Pu(IV) bound, Fe(III) bound, and mixed Pu(IV)/Fe(III) bound bovine sTf derived from small-angle X-ray scattering (SAXS). (A) Three-dimensional structural models derived from SAXS of bovine sTf binding Fe(III), Pu(IV), or a combination of both. The Pu_C_Fe_N_Tf adopts a closed conformation. The interdomain cleft in each of the open N-lobes is indicated with arrows, and the open lobe of Pu_2_Tf is superimposed on the crystal structure of open N-terminal recombinant human half apo-transferrin (PDB code: 1BP5). (**B**) Docking wireframe representations of the sTf structures onto each other reveal the closed structures of Fe_2_Tf and Pu_C_Fe_N_Tf and the mixed structures Fe_c_Tf and Pu_2_Tf. Reprinted with permission from [45]. Copyright (2011) Spring Nature Limited.

This second possibility has to do with conformational changes and their impact on protein electrostatics. Differences in the sTf coordination modality of the metal ion will result in differences in the conformation that the protein adopts after metal binding, which in turn will result in variations of the electrostatics of the metal-bound protein. Certain coordination modalities may not induce the necessary conformational change for a favorable contact with the TfR. An excellent example is the Pu(IV) ion. A small-angle X-ray scattering (SAXS) study revealed that Pu_2_Tf adopts a mixed conformation with the Pu(IV) at the C-site bound with a closed lobe and the Pu(IV) at the N-site with an open lobe [46] (Figure 13). Jensen et al. sought to examine what would happen if Pu(IV) were to bind sTf in a mixed Fe(III) metalation [46]. They synthesized the Pu_C_Fe_N_Tf and Fe_C_Pu_N_Tf complexes with the Fe(III) prebound to the protein before addition of the Pu(IV). Interestingly, the Pu_C_Fe_N_Tf complex exhibited a fully closed conformation of both lobes but the Fe_C_Pu_N_Tf complex had an open conformation at the N-lobe. Binding studies of Pu_2_Tf, Pu_C_Fe_N_Tf, and Fe_C_Pu_N_Tf with TfR revealed that Pu_C_Fe_N_Tf had the highest affinity, comparable to that of Fe_2_-sTf. The binding of Pu_2_Tf and Fe_C_Pu_N_Tf was weak even despite their mixed conformation. The Pu_C_Fe_N_Tf complex was the only Pu(IV)-bound sTf complex to deliver Pu(IV) into PC12 cells [46]. The puzzling part about this study was that although both the Pu_2_Tf and Fe_C_Pu_N_Tf complexes had the C-lobe in a closed conformation, an interlobe cooperativity does not appear to have been influential in facilitating closure of the N-lobe. However, closure of the N-lobe is not the issue here. The weak affinity to the TfR is likely owed to electrostatics. Measurements of the isoelectric points (pI) of metalated-sTf complexes performed by Brulfert and Aupiais offers insight here (Table 2) [59]. The pI of apo-sTf (6.07) and Fe_2_-sTf (5.63) are very different and can be attributed to differences in the surface charges of the proteins. The pI of the monoferric sTf complexes is not identical. The pI of Fe_C_-sTf (5.90) is higher than that of Fe_N_-sTf (5.82) and this difference may be enough to account for differences in interlobe cooperativity and TfR interactions. The pI of Pu_C_-sTf (6.02) is even higher than Fe_C_-sTf. One can speculate that the pI values of Pu_2_Tf and Fe_C_Pu_N_Tf would be much higher than that of Fe_2_-sTf and that the value of Pu_C_Fe_N_Tf would be closer to Fe_2_-sTf. Using pI as a benchmark, a feasible hypothesis is that any metalated-sTf complex having a pI in the range of Fe_2_-sTf would have a favorable interaction with TfR that would enable endocytosis. It is important to consider, nonetheless, that the coordination modality of a metalated-sTf complex will depend on synergistic anions/ligands that are present and often it is not clear whether a given synergistic anion/ligand may be physiologically relevant. At present, this limits the extent to which a full picture of the factors involving metal binding by sTf and subsequent TfR interaction can be defined in vivo.

**Figure 14.**
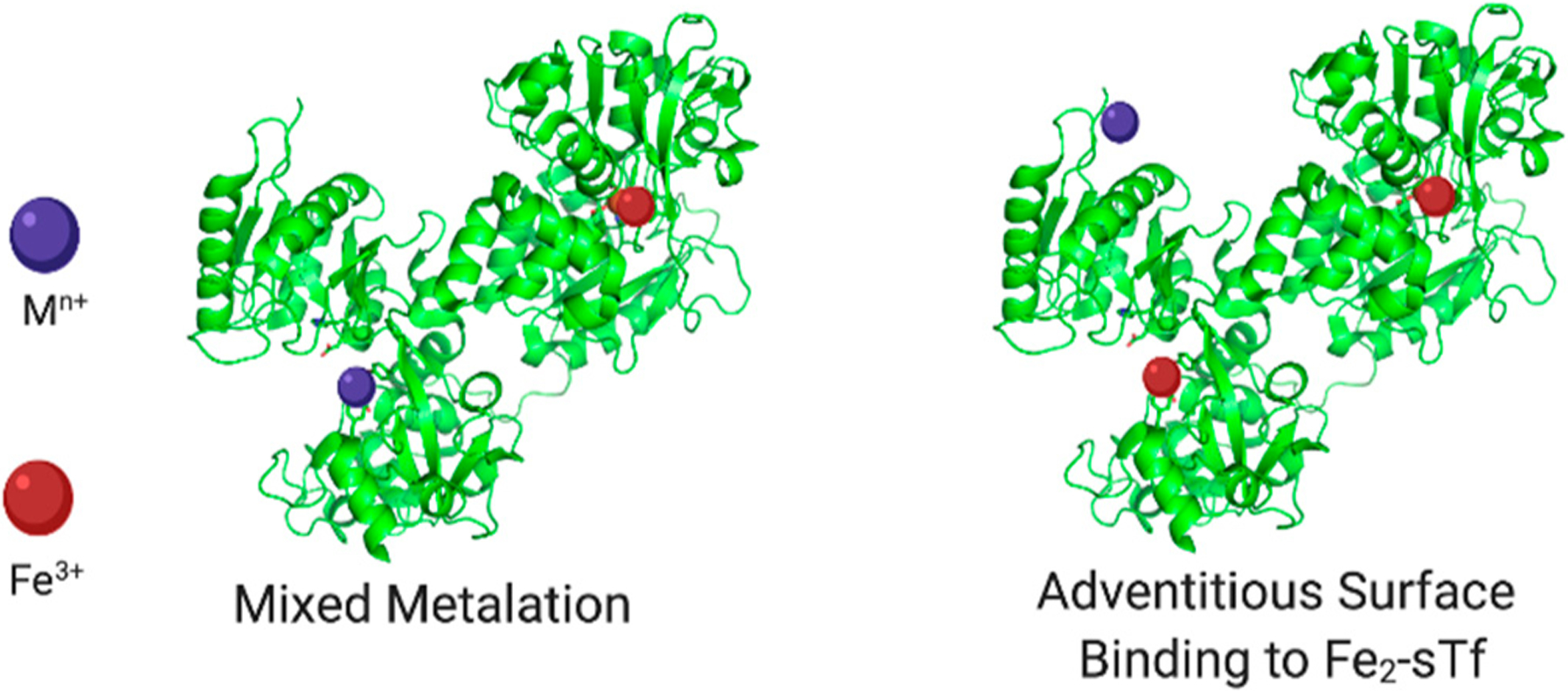
Fe(III)-bound sTf modes for nonferric metal ion cellular uptake. Created with BioRender.com.

The Pu(IV) case study highlights the relevance of Fe(III)-bound sTf in potentially contributing to nonferric metal cellular uptake (Figure 14). As a reminder, of the total sTf (30 μM), 39.2% is Fe free. It is important to be cautious and avoid stating metal free as others tend to do because possibly other metals may occupy at least a small portion of this space like manganese(II) (Mn(II)) and Ti(IV) [8,63]. The remainder consists of 11.2% Fe_C_-sTf, 22.9% Fe_N_-sTf, and 26.7% Fe_2_-sTf [9]. Of this population, monoFe(III)-sTf is the most abundant and could plausibly play a greater role in nonferric metal transport by forming mixed metalation species with stronger TfR recognition than the holo-sTf form of the nonferric metal ions. In addition, Fe_2_-sTf could also play a significant transporter role with nonferric metal ions in chelate form adventitiously coordinating to surface sites of the protein. Chromium(III) (Cr(III)), ruthenium(II/III) (Ru(II/III)), and vanadium(IV) (V(IV)) complexes have been reported to bind to the surface of Fe_2_-sTf [64–68]. Fe_2_-sTf is maximally primed for TfR delivery and it is feasible that other metals could ride on its shoulders. Therefore there are three possible modes for nonferric metal sTf entry into cells:

1. Binding canonically or noncanonically at the metal binding site;
2. Binding in a mixed metalation with Fe(III);
3. Adventitious surface binding onto Fe_2_-sTf.

It is important to note that although a metal ion may be capable of being bound by sTf, this does not mean that in vivo it will form a metal complex with the protein or even bind at its surface. An excellent example is Cu(II). It is predicted to have the highest affinity to sTf (log K_1_ 10) [4] compared to other divalent metal ions and yet sTf does not transport it in blood. Ceruloplasmin is the main Cu transporter in blood and serum albumin also contributes but to a smaller extent [69–71]. Competitive biomolecular metal binding greatly influences blood metal speciation [72–75] as does metal hydrolysis for hard Lewis acidic metals like Ga(III).

Another factor to consider pertains to the TfR-mediated endocytosis process. A metalated-sTf complex may favorably interact with the TfR and induce endocytosis. However, this does not automatically imply that the metal will actually be released into the cytosol during the process. Lay et al. have argued that sTf strictly controls metal release into the cytosol and that a number of nonferric metal ions may not be released [64,65,68]. In this capacity, sTf would play a regulatory role by protecting the body from the toxicity of certain nonferric metals. Recall that for Fe(III), reduction of the metal ion to Fe(II) is key to endosomal release. For a nonferric metal to effectively use TfR-mediated endocytosis to enter cells, it must have a molecular mechanism for endosomal release. While it would be difficult (and be beyond the scope of this work) to systematically characterize all of the nonferric metal ions that are likely to exploit this transport route, at least two molecular mechanisms are identified for endosomal release of nonredox active hard Lewis acid metals that mimic aspects of the Fe(III) pathway (Figure 9). They include:

1. Acidification, chelation, and DMT1 transport;
2. Acidification, chelation, and ionophoric transport.

In the next section, how sTf may regulate the therapeutic property and toxicity of a select group of nonredox active hard Lewis acid metals is explored.

## 5. Examining How sTf May Facilitate the Therapeutic and Toxic Properties of Redox Inert HardLewis Metal Ions

### 5.1. STf as a Vehicle for the Anti-Type 2 Diabetes V(IV) and Cr(III)

Diabetes mellitus (DM) is a multisystemic endocrine disorder characterized by persistent elevation in fasting and postprandial glucose levels resulting in disturbance of carbohydrate, lipid, and protein metabolism [76]. In DM, the body presents an absolute or relative lack of insulin (insulin-dependent IDDM, type 1) and/or does not properly respond to it, in a process also defined as insulin resistance (non-insulin-dependent NIDDM, type 2). DM may be a product of autoimmunity, obesity, or age [77]. According to the World Health Organization (June 2020), DM affects more than 422 million people around the world. Symptoms of DM frequently show up a long time after the beginning of the disease, causing chronic damage, dysfunction and failure of eyes, kidneys, nerves, heart, and vessels, finally leading to death. Hence, the main target in an antidiabetic therapy for type 1 and type 2 patients is to reach normal blood glucose levels (5 mM) and to reduce insulin resistance in order to improve metabolic control and to prevent clinical complications.

Insulin is the hormone responsible for the regulation of blood level of nutrients like glucose, amino acids and fatty acids by signaling the cellular uptake, metabolism, and transformation of these nutrients into storage macromolecules like glycogen, proteins, and lipids, respectively. During feeding, pancreatic β-cells secrete insulin in response to elevated levels of nutrients, especially glucose (postprandial hyperglycemia) and simultaneously activate insulin biosynthesis for future secretions [78,79]. The insulin receptor (IR) is an insulin-activated transmembrane protein tyrosine kinase. Following insulin binding, the IR undergoes activation by autophosphorylation at select tyrosine residues leading to the coupling and phosphorylation of several proteins (called insulin receptor substrates, IRSs). Once coupled to the phosphorylated receptor, IRSs serve as docking sites responsible for propagating insulin signaling by a phosphorylation cascade and ultimately triggering the translocation of glucose transporter 4 (GLUT4) to the cell membrane to facilitate glucose uptake [80–82]. When insulin is removed, termination of glucose metabolism occurs at several levels, including dephosphorylation of tyrosyl residues by endogenous protein phosphotyrosine phosphatases (PTPases) facilitated by phosphorylation of an active site cysteine residue [83]. In type 2 diabetes, overexpression of PTPases, in particular PTP1B, is the main cause of insulin resistance [84]. PTPases shut down the glucose uptake as they catalyze the phosphoester bond cleavage at the phosphorylated tyrosine residues of IRSs leading to the uncoupling of the IRS from the insulin receptor and downstream effectors.

Vanadium(IV) and chromium(III) (Cr(III)) have long been studied for their insulin-enhancing properties. While their essentiality in humans is debated, they exhibit tremendous therapeutic potential as anti-type 2 diabetic agents [85–92]. Both manifest their insulin-enhancing effect via related but distinct molecular mechanistic routes that are believed to be tied to the transport offered by sTf. Herein the insulin-enhancing mechanistic details of V(IV) and Cr(III) and the role of sTf in these processes are briefly reviewed.

#### 5.1.1. The Anti-Type 2 Diabetes Mechanism of Action of V(IV)

**Figure 15.**
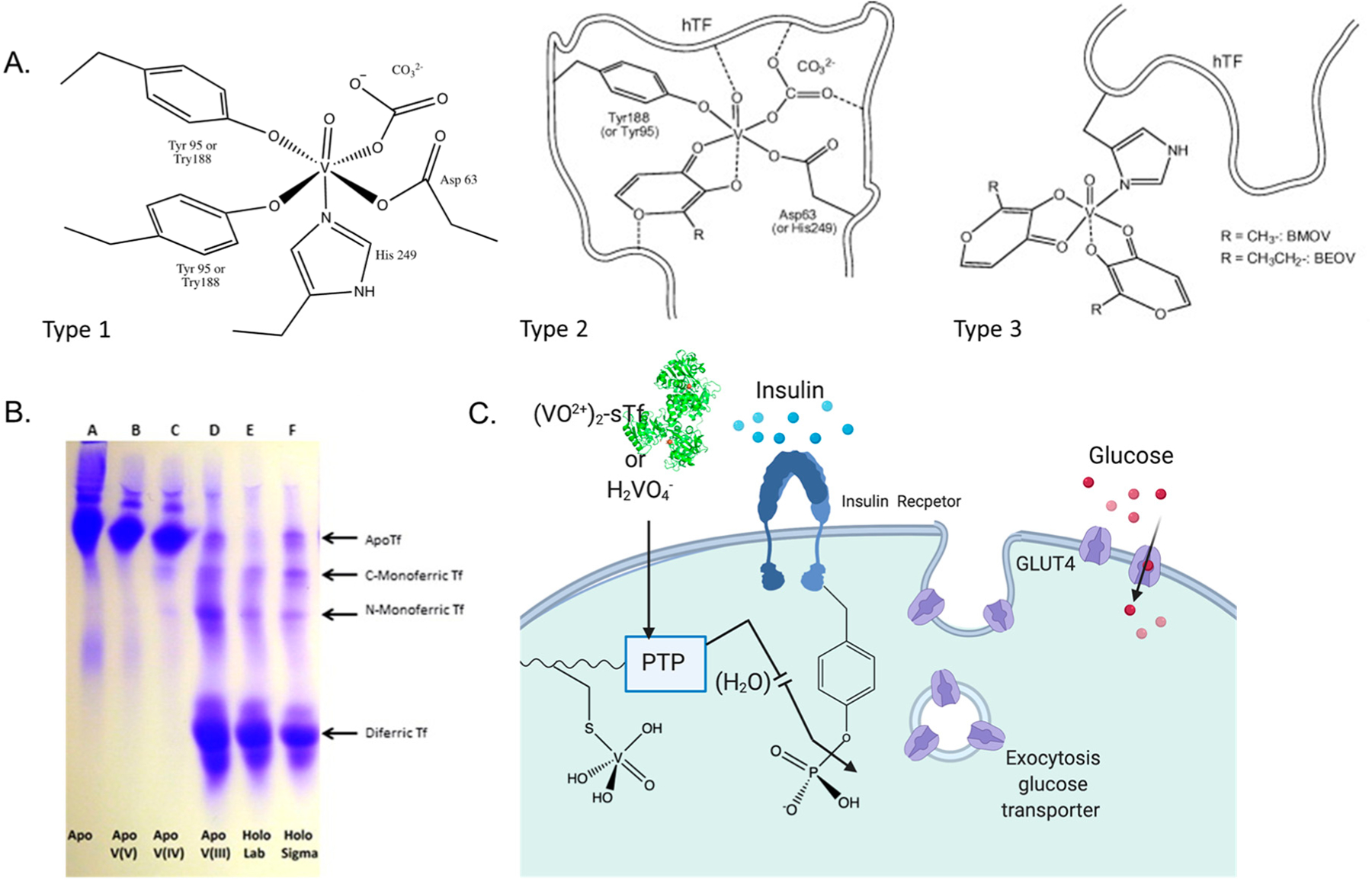
Vanadium interaction with sTf. (**A**). Three proposed types of VO^2+^ binding to sTf. Types 1 and 2 involve direct coordination to the metal binding site with and without a carrier ligand as a synergistic anion. Type 3 shows the VOL_2_ complexes binding adventitiously to a surface site of Fe_2_-sTf. (**B**). Urea-PAGE of sTf with and without V(III), V(IV), V(V), and Fe(III). Note that V(IV) should run through the gel like V(III) but the presence of EDTA in the running buffer resulted in V(IV) dissociation. (**C**). Proposed route for VO^2+^ and V(V) entry into cells and the insulin-enhancing mechanism of [VO(OH)_3_]^−^ inhibition of the PTP enzymes. (**A**,**B**) were partially adapted with permission from [67]. Copyright (2014) Elsevier. (**C**) was created with BioRender.com.

An extensive number of compounds containing the physiologically relevant V(III), V(IV), and V(V) ions have been explored as orally active insulin-enhancing therapeutics for diabetic animal models, wherein the V(IV) compounds have been found to be especially more effective as blood glucose-lowering agents [93,94]. The neutral VOL_2_ compounds bis(maltonato)oxovanadium(IV) (BMOV) [87,88,95] and bis(ethylmaltonato)oxovanadium(IV) (BEOV, Figure 15A) displayed extremely promising therapeutic potential. BEOV reached phase II clinical trials but its efficacy was deemed insufficient [89,94]. Since then no other V compounds have advanced to clinical trials. The ligands (L) in these compounds play the role of passive carriers and thus the compounds are regarded as prodrugs. Aqueous speciation studies have shown that vanadyl (VO^2+^) ions released into the cytosol will convert into the orthovanadate ([VO(OH)_3_]^−^) species. This species is an excellent phosphate mimic (H_2_PO_4_^2−^/HPO_4_^−^) and has been shown to serve as a potent PTP inhibitor [96–99]. Unlike phosphate, which binds covalently to the PTP1B active site through a 5-coordinate transition state, [VO(OH)_3_]^−^ can form a 6-coordinate highly stable complex with the phosphatase active site cysteine [98,100–102]. The PTP dissociation constant for orthovanadate is in the range of 0.4–5 μM and for phosphate is in the range of 10–30 mM [98]. The much higher affinity for orthovanadate demonstrates that it is an effective inhibitor of phosphorylation of the active site cysteine.

The main advantage of these VOL_2_ compounds is that contrary to insulin, they can be orally administered. It has been observed that as a consequence of the changing biochemical environment that the compounds encounter while traveling through the gastrointestinal tract following oral administration and then being released into the bloodstream until reaching the intracellular environment, they can undergo partial or full dissociation into free VO^2+^ ions. Nonetheless, even with the original carriers being lost during the transport, all VOL_2_ compounds display marked biological activity in the relevant concentration range 1–10 μM.

Circular dichroism (CD), electron paramagnetic resonance (EPR) (V(IV) is d^1^), and gel electrophoresis studies have demonstrated that after gut absorption and then transport through the bloodstream, most of the V in serum is bound to sTf [103–106]. Free VO^2+^ is believed to bind to both sTf metal binding sites, coordinating to all four protein residues with the oxo ligand serving as a synergistic anion. Whether the oxo group replaces carbonate or carbonate also serves as a co-synergistic anion [107] with either mono or bidenticity is not clear. This type of coordination will be referred to as Type 1 VO^2+^ coordination (Figure 15A). This coordination modality is expected to induce lobe closure. A Urea-PAGE study (Figure 15B) evaluating the binding of V(III), V(IV), and V(V) to sTf demonstrated that the metal oxidation state dramatically influences how the metal binds to the protein [67]. Proteins travel through the gel based on their relative stability. Apo-sTf does not vertically travel far in the gel whereas Fe(III)-bound sTf does, with Fe_2_-sTf traveling the most due to its high stability associated with both of its closed lobes. Monoferric sTf travels an intermediate distance. V(III) mimics the canonical Fe(III) coordination as it does not have a bound oxo group and it produces metal-STf species (monometallic and dimetallic) of comparable stability to the Fe(III) ones. V(IV), though coordinated in a noncanonical manner, can also produce metal-STf species of comparable stability to the Fe(III) ones (Figure 15B does not show this due to experimental conditions). V(V) is believed to bind with weak affinity in an open conformation. The Urea-PAGE shows the V(V)-containing sTf species to have comparable stability as apo-sTf likely because of facile V(V) dissociation [67]. There is evidence to suggest that instead of being bound by sTf, V(V) will transform into the anionic vanadate species H_2_VO_4_^−^ and use anionic channels to enter cells (Figure 15C) [68,102]. H_2_VO_4_^−^ may retain its V(V) oxidation state or be reduced to V(IV) but either form would mimic phosphate and be able to inhibit PTP1B [67].

If full ligand dissociation from VOL_2_ does not occur then the carrier ligand may participate in the stabilization of the metal ion in interaction with sTf. Sanna et al. have proposed two additional modes of VO^2+^ coordination [108]. The Type 2 mode of (VO)(sTf)(L) involves the carrier as a synergistic anion within the metal binding site. The Type 3 mode involves VOL_2_ binding adventitiously to the surface of the protein via V(IV) direct coordination to the His-N, Asp-COO^−^, or Glu-COO^−^ residues (Figure 15A) [108]. Although Fe_2_-sTf is an optimal route for cell entry, the Type 3 mode seems the least likely especially considering the extensive ligand dissociation possible through oral administration. Within the endosome, V(IV) release from sTf is predicated on VO^2+^ coordination by a chelator. Citrate and ATP are good endosomal chelator candidates because both are capable of forming labile complexes with the ion [109–111]. Citrate chelates VO^2+^ via its alpha hydroxylate and alpha and beta carboxylate moieties [109]. ATP coordinates VO^2+^ through its phosphate groups at acidic to neutral pH [111]. This particular coordination modality is expected of ATP for all hard Lewis acidic metal ions. Lay et al. have recently found that citrate may not be a sufficient chelator to remove VO^2+^ from sTf (bound at the metal binding site or at the surface of Fe_2_-sTf) within the endosome resulting in resurfacing of the ion following the endocytosis process [68]. The lability of the VO^2+^ species released from sTf is important because although the chelated metal center is relatively redox inert, the VO^2+^ ion is divalent and can be transported out of the endosome via the DMT1 [112] and be available to inhibit cytosolic PTP1B (Figure 15C).

#### 5.1.2. The Anti-Type 2 Diabetes Mechanism of Action of Cr(III)

**Figure 16.**
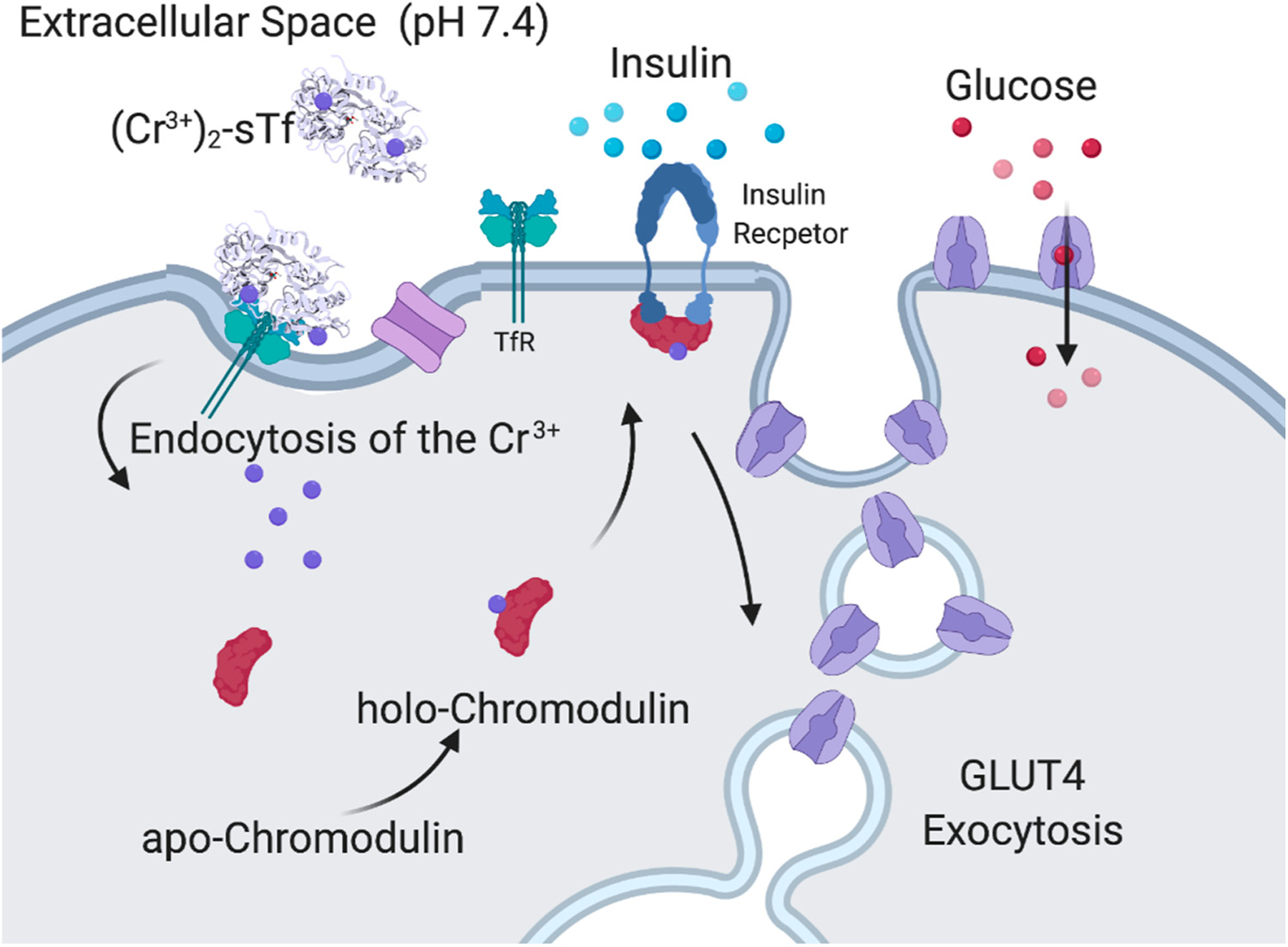
Proposed insulin-enhancing mechanism of Cr(III) facilitated by its chromodulin interaction. Created with BioRender.com.

Supranutritional amounts of Cr(III) exhibit a pharmacological effect of mediating insulin enhancement by using biomolecular pathways that are different from V(IV) [86,113]. Cr(III) absorbed from dietary sources into the body becomes 80% sTf bound [114]. It is believed to bind in the canonical modality at the metal binding site [13,91,92]. Very recently, a crystal structure for Cr(III)-bound sTf was obtained, which shows Cr(III) coordinated at the C-site, in closed-conformation, canonical modality with malonate substituting for carbonate [115]. In 1986, Yamamoto et al., discovered chromodulin, a Cr-binding oligopeptide present inside the cytoplasm and nucleus in its apo form [116]. Interestingly, apochromodulin was observed to take advantage of insulin stimulation of TfR recycling [117] to sequester up to 4 Cr(III) ions from the Cr_2_-sTf-TfR complexes entering cells. In the holo form, chromodulin binds to the insulin-bound, activated insulin receptor and is believed to further enable the phosphorylation cascade of the insulin signaling process that leads to glucose uptake (Figure 16) although there is some debate about the relevance of the chromodulin and insulin receptor interaction [64]. Cr(III) is also capable of protecting against physiological hyperinsulinemia-induced plasma membrane cholesterol accumulation and cortical filamentous actin (F-actin) loss as observed in L6 skeletal muscle myotubes [90]. The adenosine monophosphate-activated protein kinase (AMPK) is responsible for the inhibition of cholesterol synthesis via the phosphorylation of 3-hydroxyl-3-methyl-glutaryl coenzyme A reductase (HGMR). AMPK activation was found to be an important aspect of the mechanism of Cr(III) action in the insulin-resistant skeletal muscle cells. It resulted in a decrease of membrane cholesterol, which results in restoration of F-actin integrity, required for proper insulin-regulated GLUT4 translocation and glucose transport [90].

Despite the observation of the these therapeutic properties of Cr(III), there is skepticism regarding the drug potential of the metal ion because of evidence that suggests its accumulation in the body can be toxic [64]. Rather than facilitating cellular uptake of Cr(III), sTf is argued to do the opposite in order to protect the body from its toxicity. STf may decrease its cellular uptake by retaining it throughout the endocytosis process [64,91]. STf certainly has the capacity to protect the body from Cr(III), especially excess toxic amounts. The study by Lay et al. evaluates Cr(III) at concentrations well above physiological levels, which are highly likely to be toxic [64]. The affinity of sTf for Cr(III) is many orders of magnitude lower than for Fe(III), which can serve to regulate how much it can bind to the protein (Table 1). This lower affinity also makes sTf very capable of releasing Cr(III) from the endosome into cells. It has been shown that at endosomal pH, Cr(III) is rapidly released from sTf at the C-site although retained at the N-site [92]. The presence of anions such as the physiologically relevant citrate and ascorbate and not relevant EDTA can double the release rate of Cr(III) from the N-site at endosomal pH [92]. A very compelling finding is that the presence of the TfR further accelerates Cr(III) release from sTf. The metal ion should be fully released from the Cr_2_-sTf-TfR complex within 15 min, making it fast enough for physiological transport of Cr(III) [92]. The finding of Cr(III) retention by sTf-TfR by Lay et al. might be an artifact of insufficient time allowed to observe metal release and not using physiologically relevant anions in their biolayer interferometry approach [64]. It would be interesting to examine whether, in addition to citrate and ascorbate, ATP might be a relevant scavenging chelator as it is very capable of forming a Cr(III) complex. It is not clear how any Cr(III) chelate species that is released from sTf will ultimately facilitate Cr(III) escape from the endosome. In the next section, the possibility of ATP as an ionophore is discussed.

### 5.2. STf Mediation of the Cytotoxic/Antiproliferative Properties of Ti(IV) and Ga(III)

Like diabetes, cancer is a complex series of diseases that there will never be a “one size fits all” strategy against it. It is the second main global cause of death, claiming 9.56 million lives in 2017 [118]. In 2020, the American Cancer Society estimated that there would be 1.8 million new cases of cancer and 606,520 deaths in the United States alone [119]. At one point there was great interest in exploiting sTf in drug delivery strategies for anticancer applications. Due to the higher requirement of cancer cells for Fe(III) than normal cells, cancer cells have a higher expression of the TfR to increase the intake of Fe(III) from sTf to meet their greater metabolic demand [120–123]. STf could thus be used as a Trojan Horse to introduce a variety of anticancer agents. The relative success of the platinum(II)-based compounds in chemotherapy and their predominant use in the treatment regimes of 40–80% of cancer patients today [124,125] has sustained the multi-decade medicinal pursuit of other metals. Many complexes of nonplatinum metals have been designed as anticancer drugs to take advantage of the distinct chemistry exhibited by the different metals to invoke a distinct mechanism of the antiproliferative/cytotoxic effect with the possible hope of multi-faceted combinatorial therapy. A significant subset of these complexes is believed to operate via their interaction with sTf.

#### 5.2.1. STf May Facilitate the Cytotoxic/Antiproliferative Properties of Ti(IV) at High Metal Concentration

Titanium(IV) emerged as a promising anticancer agent soon after the discovery of cisplatin [126]. Two Ti(IV) compounds, titanocene dichloride (Cp_2_TiCl_2_) and budotitane, made a rapid leap to clinical trials, a total of seven between both compounds [127–131], on account of exhibiting a broad spectrum of effect against different cell lines and not demonstrating cross-resistance with cisplatin. Ti(IV) blocks human topoisomerase interaction with DNA [132] thus effectively inhibiting DNA replication. This is consistent with Ti(IV) inducing cell cycle arrest in the late S/early G2 phase. Ti(IV) is also able to induce apoptosis at any phase of the cell cycle [133]. Unfortunately, neither Cp_2_TiCl_2_ nor budotitane advanced to the clinical market due mainly to poor efficacy and formulation issues and since then no other Ti(IV) compound has entered clinical trials. The discrepancy between the in vitro and in vivo studies of the lead Ti(IV) compounds is thought to be owed to their unstable behavior in aqueous solution. Like many Ti(IV) compounds, they are extremely hydrolysis prone. At pH 7.4, they rapidly convert into polymeric hydrolyzed species [134] that are virtually devoid of all drug-like activity. This behavior suggested that the compounds are essentially prodrugs that must require a biomolecular vehicle or interaction to facilitate the biomedical properties of the metal ion.

The discovery of Ti(IV) binding by sTf [135–137] resulted in the logical hypothesis that the protein was key to the metal ion’s activity [135–139]. Rapid ligand dissociation from Cp_2_TiCl_2_ and budotitane in the presence of sTf leads to complete Ti(IV) rescue at pH 7.4 as sTf coordination results in elevated Ti(IV) blood solubility and bioavailability. Ti(IV) can bind to both of the metal binding sites as a hydrolyzed species [8,140–142]. Although the coordination modality of this species is not known, one may assume that it would be comparable to the coordination of the titanyl ion (TiO^2+^) by the ferric binding protein (Fbp) of *Neisseria gonorrhoeae*, which has a very similar metal binding site with glutamate taking the place of aspartate and phosphate taking the place of carbonate [143]. The Fbps are distantly related to the transferrins, often referred to as bacterial transferrins [144]. The Ti(IV) in this protein is only bound by the tyrosine residues and a mixture of oxo and perhaps aqua or hydroxo ligands [143]. However, sTf binding of hydrolyzed Ti(IV) in human blood seems unlikely given that Ti(IV) can rapidly be coordinated by the small anion citrate, which is present in serum at 100 μM and serves as an extracellular chelator. Speciation modeling at pH 7.4 has suggested that a Ti(IV) citrate complex (possibly Ti(citrate)_3_^8−^) would transiently be formed that then would deliver the metal ion to sTf [8,145]. More than being a chaperone for sTf binding, the citrate also serves as a synergistic anion as we have previously discussed, resulting in a semi-open conformation (Figure 11A). It is coordinated by the two tyrosines of the binding site and by carbonate and citrate as bidentate ligands. The citrate taking the place of the histidine and aspartate is probably owed to the harder Lewis acidity of Ti(IV) as compared to Fe(III). Considering that hydrolyzed Ti(IV) is capable of being bound by sTf, neither carbonate nor citrate are required for Ti(IV) binding as others have also noted [47,137]. A crystal structure has been obtained showing Ti(IV) bound at the N-site in an open conformation to only one tyrosine residue at the binding site and with only citrate as the synergistic anion (PDB code: 5H52; Figure 11B) [47]. It is important to note that as observed within the crystal lattice, there appears to be very low Ti(IV) occupancy at the N-site of this structure. At the C-site of the same structure, Ti(IV) is coordinated in a closed-conformation, canonical manner except that a malonate anion substitutes for the carbonate anion (Figure 11C) as it has been shown to do for Fe(III) binding [6,47]. Accounting for the physiological concentrations of citrate and bicarbonate (27 mM) in blood, it is expected that both anions will jointly serve as synergistic ligands for Ti(IV) coordination within the sTf binding sites producing the (Ti-citrate)_2_(CO_3_)_2_-sTf complex [8].

**Figure 17.**
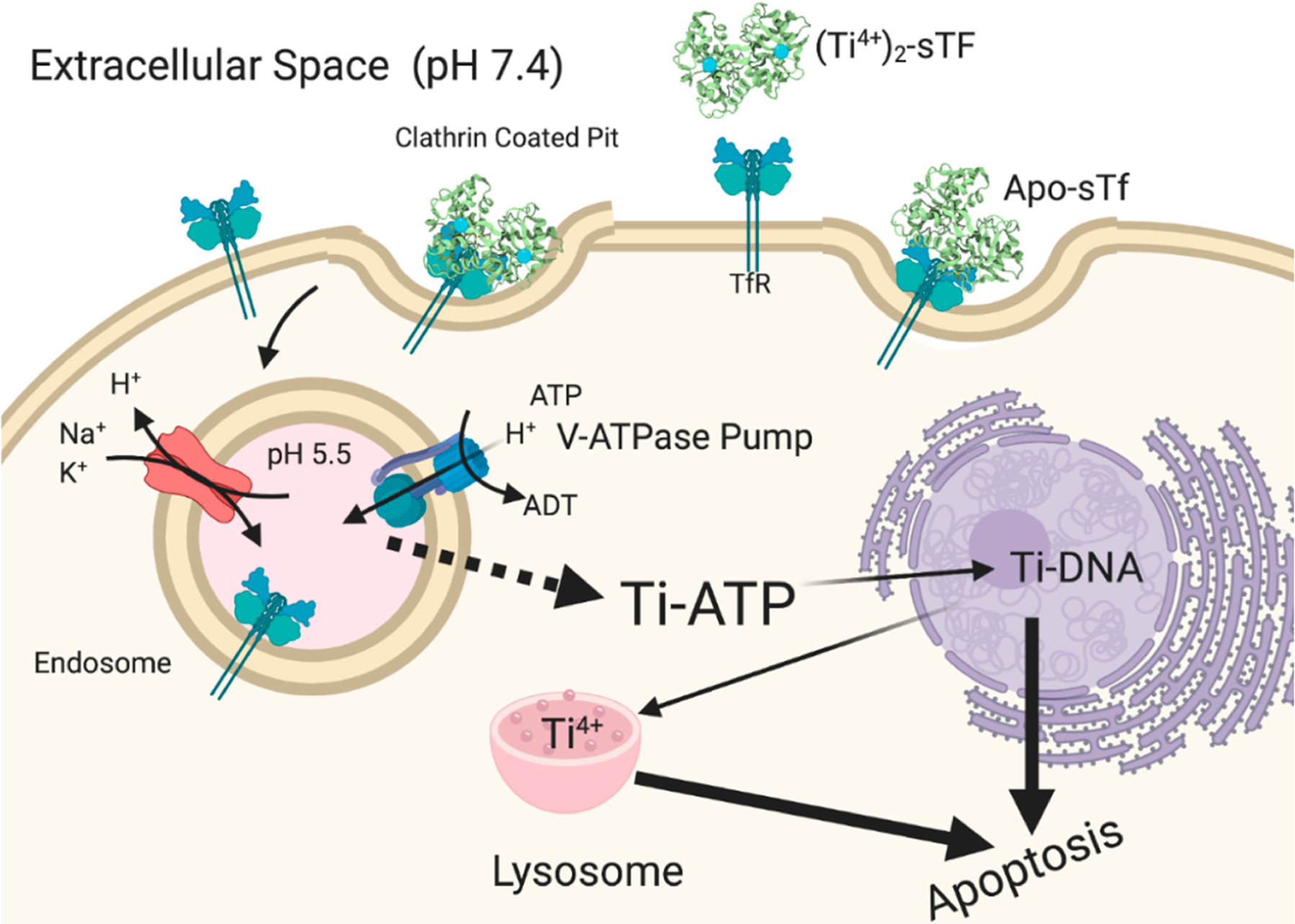
Proposed antiproliferative/cytotoxic mechanism of action of Ti(IV) from a hydrolysis prone compound like Cp_2_TiCl_2_. Created with BioRender.com.

Once Ti_2_-sTf is endocytosed (Figure 17), Ti(IV) release from sTf from the acidified endosome is not clearly understood. Two studies performed by Tinoco et al. using equilibrium dialysis show that Ti(IV) remains fully sTf bound at pH 5.5 in the presence of 100 μM citrate [8,141]. Bonvin et al., however, demonstrated by mass spectrometric direct measurement that the Ti_2_-sTf complex under the same conditions partially dissociates [49]. Sadler et al. proposed that ATP instead of citrate is the likely endosomal chelator capable of effectively scavenging Ti(IV) at this pH [137,138,146]. In addition, they proposed that ATP might serve as an ionophore to export Ti(IV) out of the endosome [137]. ATP could then transport Ti(IV) to DNA in a manner analogous to its shuttling of Fe(III) to DNA coupled with hydrolysis of the γ-phosphate group [147]. Ti(IV) accumulates in the nucleus and within cytosolic lysosomes co-localized with phosphorus as monitored by an electron-spectroscopic imaging (ESI) study of the human stomach, colon, and lung adenocarcinoma cells, isolated from xenografted mice following Cp_2_TiCl_2_ treatment [148]. Ti(IV) can bind to the phosphodiester backbone of DNA [146,149] and perhaps to phosphoproteins [150] of lysosomes, the relevance of which is not clear. Via the TfR-mediated endocytosis route coupled with further transport by ATP, Ti(IV) would be available to bind as an ion to cellular targets. DNA binding of Ti(IV) is expected to cause detrimental structural changes, which can inhibit the required interaction between topoisomerases and DNA necessary to initiate strand unwinding for DNA replication. Ti(IV) has also been shown to directly interact with topoisomerases [132,151], which might be another route for inhibiting DNA contact. Indirect secondary effects may further contribute to the inhibitory effect [152]. Increase in Ti(IV) concentration is correlated with an increase in p53 levels, a protein that participates in DNA damage repair mechanisms [133]. The biomolecular pathway for Ti(IV)-induced apoptosis, however, remains elusive.

The role of sTf in enabling antiproliferative/cytotoxic effect of Ti(IV) is well-delineated. However, this effect may be the consequence of high Ti(IV) concentration levels. The (Ti-citrate)_2_(CO_3_)_2_-sTf complex does not display antiproliferative/cytotoxic behavior at low μM concentration [153]. A similar finding was observed for Ti(IV)-bound sTf under conditions that mimic Ti(IV) release into blood from Ti-containing implants [154]. As has been proposed for sTf regulating the toxicity of Cr(III) [64], sTf may operate to attenuate the cytotoxicity of anticancer Ti(IV) complexes in synergism with citrate by inducing their dissociation [8]. This synergism might account for the lack of efficacy of Cp_2_TiCl_2_ and budotitane in clinical trials on account of fast biotransformation to the generally inefficacious (Ti-citrate)_2_(CO_3_)_2_-sTf complex in addition to the compounds’ general lack of solution stability [8,155]. It would also explain why the typical nM amount of Ti(IV) in human blood, which is sTf bound [156,157], is not believed to be toxic. In elucidating the mechanism of action of Cp_2_TiCl_2_, Christodoulou et al. did not detect any apoptotic behavior until examining the compound at mid to high μM concentrations [133]. These concentrations may define the concentration threshold at which sTf might facilitate Ti(IV) ion antiproliferative/cytotoxic properties. More recently developed titanocenyl complexes appear to operate independent of sTf and are able to putatively bind DNA intact [149]. For a Ti(IV) complex to be an effective anticancer agent at low dose administration, it is highly recommended that it have no interaction with sTf and that it use alternative cell uptake routes.

#### 5.2.2. The Cytotoxic/Antiproliferative Properties of Ga(III) Is Owed to Its Biomimicry of Fe(III)

When researchers discovered that radioactive gallium (^67^Ga) could be used to localize malignant tumors in vivo, they began to be interested in the mechanism behind the uptake of ^67^Ga by the malignant cells. As previously stated, Ga(III) is able to mimic the bioinorganic coordination chemistry of Fe(III) except its redox activity. It shares similar properties such as its ionic radii (0.620 Å for 6-coordinate complexes) and affinity constants to biological ligands, such as sTf (Table 1) [56,58,158]. STf is central to the cytotoxic/antiproliferative properties of Ga(III)-containing complexes [159]. The ability of Ga(III) to interact with sTf is highly environment dependent regardless of the high affinity of their interaction and this can greatly affect the biological impact of the metal ion.

Through transport by sTf, Ga(III) is able to inhibit cellular proliferation by blocking Fe bioavailability or inhibiting biomolecular coordination of Fe, which is required for proper cellular functioning [160–162]. Fe is an important component of cellular division and metastasis of cancer cells [163]. Ribonucleotide reductase (RNR) is a Fe-dependent enzyme that is vital to the formation of deoxynucleotides, which are essential for DNA replication. The enzyme is composed of two subunits: M1 and M2. The M1 subunit contains the substrate (oxynucleotide-diphosphate) and effector binding sites, while M2 contains a binuclear Fe center and a tyrosyl free radical [56,58,158]. The M1 subunit is present during all of the phases of the cell cycle while the M2 unit is only present between the G1 and S phase of the cell cycle, when DNA replication occurs [56]. The activity of the enzyme depends on the formation of the tyrosyl free radical, generated by RNR binding of the Fe ions [158]. Decreasing levels of the LIP would attenuate RNR activity. The activity of the enzyme can be monitored by electron paramagnetic resonance (EPR) spectroscopy because the tyrosyl free radical produces a characteristic g ~2 signal [56]. The intensity of this signal serves as a good indicator of RNR activity. Ga(III) treatment of cells is able to block cellular uptake of Fe(III) via TfR-mediated endocytosis and, consequently, decrease RNR activity. A comparable situation was observed by Sadler et al. with Ti_2_-sTf blocking cellular uptake of Fe_2_-sTf [137]. The effect of Ga(III) can be reversed with Fe(III) supplementation [159].

There are two other ways in which Ga(III) may also be able to inhibit RNR activity. One proposed route is Ga(III) binding at the bisiron center of M2, which would serve as a barrier that prevents Fe(III) binding. Cell-free experiments have been performed in order to demonstrate if Ga(III) can interact with RNR and the results show that Ga(III) is able to displace Fe(III) from the M2 unit and thus inactivate RNR [158,161]. This particular interaction may not be feasible within cells unless high Ga(III) intracellular levels can be achieved. Another proposed route of Ga(III)-induced RNR inhibition is by competitive inhibition of the binding of the nucleotide substrates. Given that ATP stably binds Ga(III), it was proposed that Ga(III) could be coordinated by nucleotide substrates [164,165]. Cell-free experiments have demonstrated that Ga(III) does competitively inhibit substrate binding by RNR by modifying them via coordination so that they are unable to bind within the catalytic site [166]. Ga(III) binding of nucleotides and Ga_2_-sTf blocking of Fe(III) cellular uptake are believed to be Ga(III)’s prime methods of attack [166,167].

In lymphoma cells, Ga(III) in the form of Ga(III) nitrate (Ga(NO_3_)_3_) can induce apoptosis through the mitochondrial pathway. This process involves the activation and translocation of BAX, which is a proapoptotic protein, into the mitochondrial membrane. This leads to the release of cytochrome c from the mitochondria and the activation of executioner caspase [56,161]. Ga(III) is also able to promote oxidative stress in cells possibly by interfering with intracellular Fe regulation.

A clinical trial was performed to evaluate the efficacy of Ga(NO_3_)_3_ for the treatment of advanced bladder cancer in seven patients [162,168]. A high-dose administration (300 mg/m^2^/day given as a constant infusion daily for 7 days and repeated every 3 weeks) proved to be very effective as it resulted in significant tumor mass decrease and stable disease. As a follow-up, blood samples taken from the patients before, during, and after the continuous infusion were evaluated. Findings demonstrate that Ga(III) interferes with Fe(III) metabolism. All seven patients developed hypochromic microcytic anemia. Roughly the same amount of Ga(III) as Fe(III) was found to be associated with sTf. There was acute tissue Fe depletion or poor tissue Fe uptake during the Ga(III) infusion resulting in the formation of a non-transferrin bound labile Fe pool, which can become quite toxic as it can generate excessive ROS levels. There was also a 3.3-fold increase in zinc(II) (Zn(II)) protoporphyrin levels, which is an indicator of Fe deficiency. This deficiency was correlated with an increased TfR expression, as the body seeks to capture whatever available Fe is present in blood. Hypothetically, if the blood levels of Ga(III) were to exceed the micromolar levels of Fe(III), it would outcompete Fe(III) for sTf binding sites and become very toxic.

Collectively, Ga(III) is able to interfere with the bioavailability and functionality of Fe(III) within cells but typically Ga(III) salts and complexes require relatively high concentrations (mid micromolar or higher) to be efficacious [56,158,161,162,169]. The potency of Ga(III) improves if administered as a sTf complex [159] presumably due to bypassing relatively slow kinetics of sTf coordination of Ga(III). Part of the issue could be what was previously discussed, that the Ga_2_-sTf complex has a moderate affinity to the TfR and thus more of the complex is required for a pronounced effect to be exhibited. While it is not entirely clear how Ga(III) is able to escape the TfR mediated endosome, it is quite possible that the moderately lower affinity of Ga(III) to sTf enables it to be readily chelated by ATP. As already mentioned, ATP is able to form a Ga(III) complex and previous studies have demonstrated the capacity for ATP to serve as a biomolecular transporter for Ga(III) [164,165,170].

Direct coordination to the sTf metal binding sites may not be an optimal way for a metal to achieve its therapeutic effect as seen with Ga(III) and Ti(IV). Often, the link to sTf and the anticancer activity of a metal is dubious. A very good example is the antimetastatic Ru(III)-based compounds NAMI-A and KP1019/1339 [171–174]. Both compounds are quite labile and have the capacity to fully dissociate and deliver Ru(III) to both sTf metal binding sites [175,176]. KP1019/1339, which is less labile, can also behave like the antidiabetic vanadyl compounds BMOV and BEOV and bind to surface sites of Fe_2_-sTf. Regardless of these interactions, in vitro and in vivo studies suggest that there is an insignificant interaction between these compounds and sTf. Instead these compounds interact extensively with human serum albumin (HSA), the predominant blood protein that is present at 15–20 times the levels of sTf [65,177–179]. STf binding of the Ru(III) ion from anticancer compounds may serve to attenuate their activity [179] enabled by the poor affinity of the Ru(III)-bound sTf complex to the TfR [177]. While sTf has the capacity to facilitate the anticancer potential of different nonferric metal ions, questions remain regarding the feasibility of this regulatory dynamic for clinical application.

### 5.3. STf May Facilitate a Lifelong Exposure to Radioactive Pu(IV)

That sTf can help transform otherwise nontoxic metals into cytotoxic species, especially at high concentrations, is without question. STf has also been associated with enabling several toxic metal ions to persist in our bodies. Aluminum(III) (Al(III)) binding to apo-sTf or Fe(III)-bound sTf [175,180–182] has been controversially thought to be a contributing factor to diseases of the central nervous system and poor responses to vaccines [183–185] and directly impact on bone health by competing with calcium(II) (Ca(II)) in the mineralization process leading to calcium deficiency and osteomalacia [186]. STf has also been connected with the biodistribution of toxic lanthanides and actinides that seep into the environment from the production of nuclear materials in nuclear power industries [54,55,59,60,187–189]. Studies focused on the interaction of sTf with the actinide Pu afford clues into physiological cellular acquisition pathways by which sTf can facilitate metal toxicity.

Pu is a highly redox active synthetic element with all of its isotopes radioactive. Approximately 60 tons of spent Pu is produced annually with 1000 tons in storage and very little of it recycled. As a result, it greatly accumulates as a heavy-metal waste product [190]. There is much interest in understanding the long-term effects of acute and chronic exposure to the metal given that it is a severe environmental toxin. Pu that enters the body becomes principally localized in the liver and skeleton and is tightly held for decades [46]. The fissionable Pu isotopes Pu^239^ and Pu^241^ have half-lives of at least decades, posing a lifetime risk of exposure to the radioactive energy of alpha particles emitted during decay [190].

In serum, 70–90% of Pu released into the body becomes sTf bound [188,191]. As it is for Ti(IV) [142,155], sTf is likely responsible for the biodistribution and retention of Pu. A MD study examining sTf coordination of Pu(IV), the most important oxidation state physiologically [60,188], suggests that Pu(IV) can bind to all four protein residues of the metal binding site, carbonate, and two to three water molecules, producing a complex of coordination number 8 or 9. This noncanonical coordination modality exists within a closed lobe conformation [55]. Experimental studies, however, have shown that Pu(IV) binding does not induce a globally closed protein conformation [46,59,60]. This is not surprising given the large 6 to 8-coordinate ionic radii (0.86–0.96 Å) [52] of Pu(IV) can require significant structural accommodation during coordination and the metal ion tends to form complexes with high coordination numbers [5]. Jensen et al. made the fascinating discovery that Pu(IV) may hijack Fe(III)-bound sTf for cellular entry [46]. As described previously, the mixed metalation complex Pu_C_Fe_N_Tf exists as a fully closed conformation protein, which has a high affinity for the TfR and is able to facilitate cellular entry of the metal ion. It appears as though the prebinding of Fe(III) to the N-site induces a large cooperative effect on the conformation of the C-lobe that primes it for binding Pu(IV) in a closed conformation, perhaps with the coordination modality proposed by Mishra et al. [55]. The full molecular details of Pu(IV) coordination are not known. Endosomal release of Pu(IV) into the cytoplasm is expected to be triggered by the release of Fe(III) from the N-site. This release and the associated opening of the N-lobe is expected to structurally impact the C-lobe by partially opening it and weakening the C-site affinity for Pu(IV). A chelator like ATP can then transport Pu(IV) into the cytosol. ATP is able to form stable actinide complexes [192]. The Pu_C_Fe_N_Tf complex does not exhibit any antiproliferative/cytotoxic effect against PC12 cells where it was shown to have significant cellular uptake. Short term health effects may not be likely but the long term radioactive decay of the metal in the body is a warranted concern [46].

## 6. Engineering sTf for Delivery of Metal-Based Biomedical Tools for Cancer Applications

**Figure 18.**
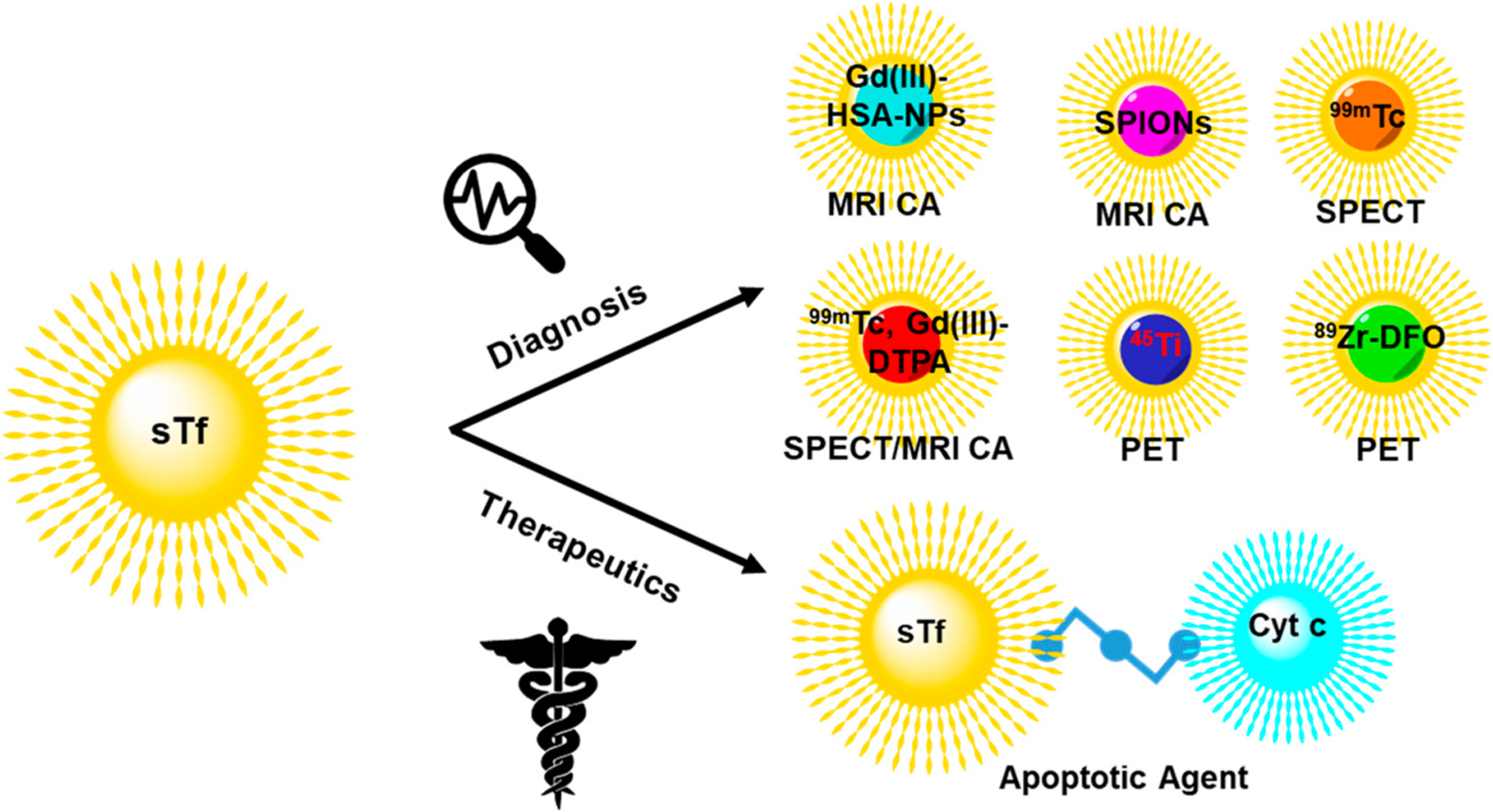
Engineering of sTf for delivery of select metal-based diagnostic and therapeutic biomedical tools for cancer applications.

Due to the overexpression of the TfR on cancer cells [120–123,193], sTf is a prime biological agent for engineering as a transport system. Different molecular cargo has been appended to sTf for diverse applications in cancer diagnosis and therapeutics. Herein a few metal-based applications are surveyed to highlight the diversity of biomedical tools built from sTf for improving human health (Figure 18).

STf conjugates of metal compounds and metal-bound sTf complexes have been widely developed to achieve an efficient targeted delivery of the metal component to tumors for diagnosis and therapy. Diagnostic agents include magnetic resonance imaging contrast agents (MRI CAs) and single photon emission computed tomography (SPECT) and positron emission tomography (PET) imaging probes. For these applications, metal release into the cytosol is not a requirement. Gadolinium(III) has historically served as an excellent T_1_ type MRI CA because of its unpaired seven electrons. Gadoversetamide is a Gd(III)-based MRI CA (best known as OptiMARK) and is a gold standard in the field. However, it like other Gd(III) compounds have been found to not be particularly stable in solution, resulting in ligand dissociation and the formation of the aquated Gd(III) ion [Gd(H_2_O)_9_]^3+^, which is toxic to cells and causes nephrogenic systemic fibrosis (NSF) [194]. Direct binding of Gd(III) to apo-sTf was initially considered by Zak et al. to try to develop a stable delivery system for the metal ion [195]. However, Gd(III) was found to only be able to bind to the C-site with low affinity (K = 6.8 × 10^6^ M^−l^). Its binding was completely blocked when studied in the presence of blood levels of bicarbonate, which suggests that in vivo a Gd(III)-sTf complex would not exist [195]. Korkusuz et al. took an alternative approach by preparing a stable conjugate between human serum albumin nanoparticles (HSA NPs) and the Gd(III) complex of diethylenetriamine pentaacetate (Gd-DTPA) and covalently coating it with Fe_2_-sTf (Gd-HSA-NP- Fe_2_-sTf) [196]. The dual protein approach was found to be an efficient MRI CA and could have applications for brain imaging [196]. Fe_2_-sTf has also been conjugated to the T2 type MRI CA superparamagnetic iron oxide nanoparticles (Fe_2_-sTf-SPIONs) by Jiang et al. for targeting and imaging of brain glial tumors in rat models [197]. Their work showed efficient and specific internalization of Fe_2_-sTf-SPIONs in C6 glioma cells along with prolonged retention and detection.

STf has also been adapted for SPECT imaging of tumors. Smith et al. synthesized a ^99m^Tc-labeled sTf complex by reducing disulfide bonds of Fe_2_-sTf so that the thiols groups could coordinate the ^99m^Tc in its +3 or +4 oxidation state. They assessed its suitability in imaging small and large tumors of xenografted mice [198]. The reported mean tumor uptake (of the injected dose per gram of tissue) in small tumor xenografted mice was 6% and in large tumor xenografted mice was 1.7% with the mean tumor/blood ratios of 2.7 and 1.7, respectively. This suggested that ^99m^Tc-labeled sTf displayed promising potential as a SPECT agent. In an interesting study, Gu et al. developed a ^99m^Tc labeled sTf-Gd-DTPA tumor-targeting dual-modal probe with magnetic and radioactive properties [199]. The probe exhibited high specificity and sensitivity in the noninvasive detection of TfR overexpressed breast tumor cells and in xenografted mice by SPECT/MRI. A higher uptake of radioactivity in the liver and kidneys was observed, suggesting the metabolism and renal clearance of the probe.

In recent years non-ferric metal labeled sTf has been explored for PET imaging. Evans et al. developed an ^89^Zr-labeled sTf radiotracer by conjugating Fe_2_-sTf with the ^89^Zr(IV) desferrioxamine B complex [200]. They used it to image tumor burden in the brain of mice inoculated with subcutaneous xenografts of TS543 glioblastoma cells [200]. ^45^Ti has also been studied as a suitable PET agent because it has a half-life of 3.09 h, low maximum positron energy, and high positron emission branching ratio. Welch et al. utilized the ^45^Ti radioisotope to understand the biodistribution of ^45^Ti-labeled Tf, in which ^45^Ti(IV) was directly bound at the metal binding sites [201,202]. PET imaging of BALB/c mice implanted with mammary carcinoma tumors (EMT-6) showed ^45^Ti localization at the tumors, with uptake up to 24 h after injection [201,202].

STf has also been engineered to deliver metal-based proteins for cancer applications. Cytochrome c (Cyt c) is a heme protein that plays a significant role in maintaining cellular homeostasis through type II apoptosis [203]. There has been great interest in exploiting the apoptotic property of Cyt c for the development of an anticancer agent but the protein is not cell-permeable. To overcome the permeability limitation, Saxena et al. developed a Cyt c-Fe_2_-sTf conjugate using a crosslinker (Sulfo-LC SPDP) [204]. The soluble conjugate displayed an enhanced uptake in cancer cells (such as A549 lung cancer cells) relative to noncancerous cells and reasonable antiproliferative potency likely owed to retaining its ability to induce caspase 3-mediated apoptosis [204].

## 7. Conclusions

This work broadens the understanding of how sTf can bind nonferric metal ions and regulate their bioavailability, bioactivity, and impact on human health. It challenges preconceived notions regarding metalated-sTf interactions with the TfR that facilitate the endocytosis process. By exploring recent X-ray structural studies, diverse modalities for noncanonical sTf coordination of metal ions have been identified that exhibit a wide range of associated protein conformational changes. A closed globular protein conformation has been determined not to be the sole determining factor for TfR recognition of metalated-sTf but rather appropriate electrostatic changes appear to be a major contributor. Further studies are required to unravel key electrostatic changes that enable favorable metalated-sTf and TfR contacts to trigger the onset of endocytosis.

Speciation analyses elucidate the physiological relevance of certain metalated-sTf complexes especially in the context of their potential for delivering nonferric metal ions into cells via TfR-mediated endocytosis. Ferric-bound sTf emerges as a major player in this transport process. Nonferric metal ions are revealed to be bound by sTf in three modes: exclusively to the two metal binding sites, in a mixed metalation with Fe(III) at these sites, or adventitiously to the protein surface of Fe_2_-sTf. One or more of these modes of binding may be relevant for different nonferric metals especially in the chelate form. Regardless of the mode of binding, two major pathways are defined for endosomal release of nonferric metal ions into the intracellular environment: DMT1 transport or ionophoric transport.

By focusing on non-redox active hard Lewis acidic metal ions, we examine how sTf-TfR delivery and endosomal release of the metal ions can dictate their therapeutic potential and toxicity. STf can feasibly participate in the distinct anti-Type 2 diabetes mechanisms of action of V(IV) and Cr(III). However, although sTf can facilitate the antiproliferative/cytotoxic properties of Ti(IV) and Ga(III), targeting the binding of these metal ions to sTf may not be an optimal anticancer strategy because of competitive biomolecular interactions and hydrolysis propensity. Additionally, sTf can operate to attenuate the toxicity of these potentially therapeutic metals. As seen with the Pu(IV) case study, sTf can also exhibit the opposite effect by enabling metal toxicity. Numerous efforts have been made to exploit the sTf-TfR cellular transport system especially for the development of biomedical tools including metal-based ones for cancer applications.

With this study, we highlight new avenues of research to evolve our understanding of metal binding and regulation by sTf, further expanding the significance of this protein to the human body.

## Figures and Tables

**Table 1.** M^*n*+^-sTf affinity and detection of M^*n*+^-sTf complexes with TfR.

Metal Ion (M^*n*+^)	M^*n*+^-sTf (log K_1_)	M^*n*+^-sTf-TfR Complex Detected?
Ti^4^+	35.8^a^	Yes^b,c^
Pu^4^+	25.0^d^	Weak^e,f^
T_C_^4^+	23.0^b^	Yes^b^
Fe^3+^	22.5^c^	Yes^b,c,e,f^
Co^3+^	21.5^b^	Yes^b,g^
Ga^3+^	20.3^h^	Moderate^b,e,g^
Bi^3+^	19.4^b^	Yes^b,g^
Th^4^+	18.65^e^	Yes^e^
In^3+^	18.5^c,e^	Yes^c^
Cr^3+^	17^i^	Yes^c^
Al^3+^	13.8^b^	Weak^b,g^
UO^2^+	13^j^	Yes^e,g^

a Taken from Ref. [8];

b taken from Ref. [48] and references within;

c taken from [49] and references within;

d taken from Ref. [59];

e taken from Ref. [60];

f taken from Ref. [46];

g taken from Ref. [61];

h taken from Ref. [58];

i taken from Ref. [4];

j taken from Ref. [62] and references within.

**Table 2.** The isoelectric points (pI) of apo-sTf and different M-sTf complexes. The pI values were taken from Ref. [59]. (N.D. = Not determined).

Species	pI
Apo-sTf	6.07 ± 0.02
Fe_c_-sTf	5.90 ± 0.02
Fe_N_-sTf	5.82 ± 0.02
Fe_2_-sTf	5.63 ± 0.02
Pu_c_-sTf	6.02 ± 0.02
Pu_N_-sTf	N.D.
